# Impact of the terrestrial-aquatic transition on disparity and rates of evolution in the carnivoran skull

**DOI:** 10.1186/s12862-015-0285-5

**Published:** 2015-02-04

**Authors:** Katrina E Jones, Jeroen B Smaers, Anjali Goswami

**Affiliations:** Center for Functional Anatomy and Evolution, Johns Hopkins University, Baltimore, MD USA; Department of Anthropology, Stony Brook University, Stony Brook, New York, NY 11794-4364 USA; Department of Genetics, Evolution & Environment, University College London, Gower Street, London, WC1E 6BT UK; Department of Earth Sciences, University College London, Gower Street, London, WC1E 6BT UK; Department of Organismic and Evolutionary Biology, Museum of Comparative Zoology, Harvard University, 26 Oxford Street, Cambridge, MA 02138 USA

**Keywords:** Disparity, Carnivora, Fissiped, Pinniped, Cranial morphology, Radiation, Ecological transition, Aquatic mammal

## Abstract

**Background:**

Which factors influence the distribution patterns of morphological diversity among clades? The adaptive radiation model predicts that a clade entering new ecological niche will experience high rates of evolution early in its history, followed by a gradual slowing. Here we measure disparity and rates of evolution in Carnivora, specifically focusing on the terrestrial-aquatic transition in Pinnipedia. We analyze fissiped (mostly terrestrial, arboreal, and semi-arboreal, but also including the semi-aquatic otter) and pinniped (secondarily aquatic) carnivorans as a case study of an extreme ecological transition. We used 3D geometric morphometrics to quantify cranial shape in 151 carnivoran specimens (64 fissiped, 87 pinniped) and five exceptionally-preserved fossil pinnipeds, including the stem-pinniped *Enaliarctos emlongi*. Range-based and variance-based disparity measures were compared between pinnipeds and fissipeds. To distinguish between evolutionary modes, a Brownian motion model was compared to selective regime shifts associated with the terrestrial-aquatic transition and at the base of Pinnipedia. Further, evolutionary patterns were estimated on individual branches using both Ornstein-Uhlenbeck and Independent Evolution models, to examine the origin of pinniped diversity.

**Results:**

Pinnipeds exhibit greater cranial disparity than fissipeds, even though they are less taxonomically diverse and, as a clade nested within fissipeds, phylogenetically younger. Despite this, there is no increase in the rate of morphological evolution at the base of Pinnipedia, as would be predicted by an adaptive radiation model, and a Brownian motion model of evolution is supported. Instead basal pinnipeds populated new areas of morphospace via low to moderate rates of evolution in new directions, followed by later bursts within the crown-group, potentially associated with ecological diversification within the marine realm.

**Conclusion:**

The transition to an aquatic habitat in carnivorans resulted in a shift in cranial morphology without an increase in rate in the stem lineage, contra to the adaptive radiation model. Instead these data suggest a release from evolutionary constraint model, followed by aquatic diversifications within crown families.

**Electronic supplementary material:**

The online version of this article (doi:10.1186/s12862-015-0285-5) contains supplementary material, which is available to authorized users.

## Background

Understanding factors which influence tempo and mode in evolution is an important theme in evolutionary biology [1-3]. One factor which may influence the evolutionary patterns is ecology [4,5]. In particular, the ‘adaptive radiation’ model of Simpson (1944) suggests that when organisms enter a new adaptive zone, that is a niche with relatively few competitors, there will be an initial burst in evolutionary rate [6,7]. This ‘early burst’ may be followed by a slowing of morphological diversification as the niche begins to become filled [2,8,9]. This model provides a potential link between ecological transitions and evolutionary rate.

Support for adaptive radiations has been found using empirical data [10-17], the best-known examples including Darwin’s finches [18-20], Hawaiian silverswords [21] and African lake cichlids [22,23]. Further, in terms of marine mammals, cetaceans underwent a rapid increase in body size disparity early in the clade’s history despite the lack of a rapid initial taxonomic diversification [24].

The mammalian order Carnivora is an ecologically and taxonomically diverse group of mammals which have been the source for many studies of morphological variation, though they display relatively low cranial disparity relative to other mammal orders [25-33]. Arguably the largest ecological transition in carnivoran evolution was the shift from terrestrial to aquatic lifestyle in the evolution of the Pinnipedia (seals, sea lions and walruses) [34]. This study investigates how this extreme ecological shift has influenced disparity (morphological diversity) and rates of evolution in pinniped skulls, in comparison to their fissiped relatives. The Carnivora provides an ideal case study for the influence of the terrestrial-aquatic transition on disparity and rates of evolution because both aquatic pinniped carnivorans (seals, sea lions, and walruses) and terrestrial (fissiped) carnivorans (dogs, bears, weasels, cats, hyaenas, mongooses and allies) have a large extant taxonomic diversity [33,35-37]. Moreover, pinnipeds are less divergent from their closest living fissiped relatives in cranial morphology than other marine mammal groups (e.g., cetaceans or sirenians) [38] and thus may be directly compared with fissipeds. Though increased rates of body size evolution in pinnipeds relative to fissipeds were not supported [39], rates of evolution in the cranium have not been compared.

**Figure 1:**
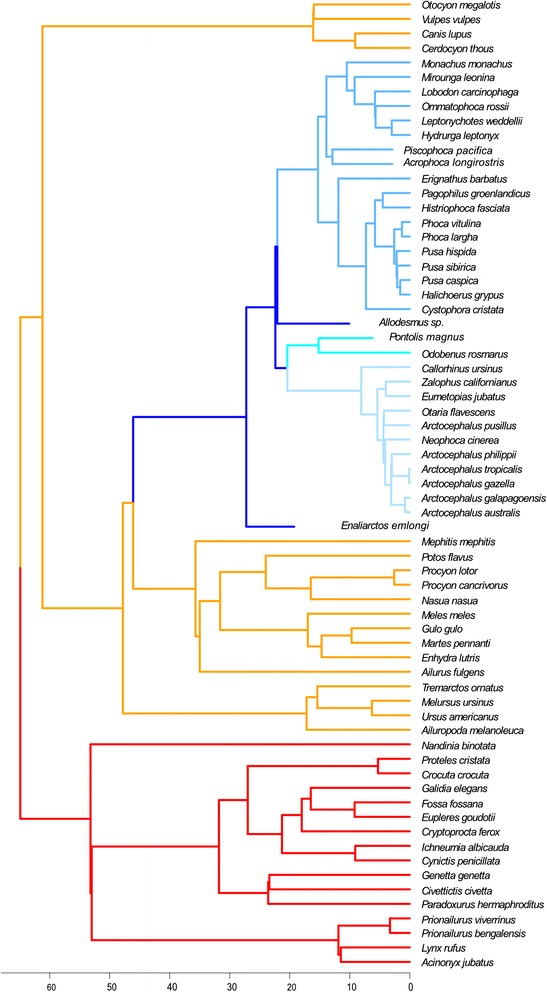
**Composite phylogeny used in this study.** Extant relationships and branch lengths from [40], placement of fossils according to [41]. This shows the Otarioidea topology, with Odobenidae as sister taxon to the Otariidae. Analyses were also run on the same tree but with a Phocidae-Odobenidae sister grouping, following the Phocomorpha hypothesis. Branch colors: Feliformia, orange; non-pinniped Caniformia, red; stem pinnipeds and allodesmines, dark blue; Phocidae, mid-blue; Odobenidae, teal; Otariidae, pale blue.

In order to test hypotheses of cranial evolution in the Carnivora, we employed analyses that work within a phylogenetic framework. The phylogenetic relationships of fissiped and pinniped carnivorans used here are shown in Figure 1 [40,41]. There are 21 genera and 34–36 species divided into the three pinniped families: Phocidae (seals), Otariidae (fur seals and sea lions) and Odobenidae (walruses), which diverged ~29ma [40,42-44]. Molecular data generally indicate that odobenids are most closely related to otariids, forming the Otarioidea (Figure 1) whereas morphological data links the odobenids with phocids in the clade Phocomorpha (Figure 1) [37,44,45], so both hypotheses were employed here. The most basal pinnipeds were the enaliarctines, a stem radiation first known from California around 28ma [45], of which one representative fossil with excellent cranial preservation is included in this study (see Methods).

The paraphyletic fissipeds are a group of mainly terrestrial (including arboreal and fossorial) carnivorans which consist of ten families, 105 genera and over 241 species (Figure 1); [46]. Pinnipeds are caniform carnivorans, and molecular and morphological evidence support the placement of pinnipeds within the arctoids (bears, raccoons, weasels, and allies). Within Arctoidea, there has been long-standing disagreement over whether musteloids or ursids are the sister group of pinnipeds (Figure 1) [35,36,45]. Most [35,40], but not all [37,47], recent evidence supports a closer relationship between musteloids and pinnipeds. Pinnipeds were also previously thought to be diphyletic, with otariids linked to ursids and phocids linked to mustelids [48,49]. However, a monophyletic origin of pinnipeds is now well supported by both morphological and molecular evidence [36,50].

To investigate the influence of the terrestrial-aquatic transition on morphological diversity, we tested evolutionary models on the carnivoran phylogeny. By combining a phylogeny with species’ information, statistical models can be used to infer the evolutionary past [51]. Such statistical models use specific parameters to determine how traits change in phylogenetic space. The most commonly used statistical model is Brownian Motion (‘BM’), which assumes that traits evolve in each instant of unit of time with a mean change of zero and unknown and constant variance. Within BM, the evolution of a continuous trait *X,* along a branch over time increment *t,* is quantified as dX(*t*) = *σ*d*B*(*t*), where *σ* constitutes the magnitude of undirected, stochastic evolution (*σ*^2^ is generally presented as the BM rate parameter) and d*B*(*t*) is Gaussian white noise.

Although generally agreed to be an unrealistic assumption for most analyses, the advantage of BM is that it is mathematically tractable. Recent phylogenetic comparative methods have continued using BM as a baseline model, but incorporate additional parameters to reflect more nuanced assumptions about the evolutionary process. Recent advances include the development of methods based on Ornstein-Uhlenbeck (‘OU’) assumptions. The OU model incorporates stabilizing selection as a constraint and quantifies the evolution of a continuous trait *X* as d*X*(*t*) = *α*[*θ* – *X*(*t*)]d*t* + *σ*d*B*(*t*), where *σ* captures the stochastic evolution of BM, and *α* determines the rate of adaptive evolution towards an optimum trait value *θ* [52,53]. This standard OU model can be modified into multiple-optima OU models that allow optima to vary across the phylogeny [54]. In these implementations, the optima are defined *a priori* to allow testing of alternative parameterizations and therefore alternative biological hypotheses [54]. An added advantage of these OU model fitting approaches is that they allow proper multivariate models rather than fitting variables one at a time [55]. Other recent methods do not fix the number of shifts or their locations on the phylogeny, but instead implement algorithms that estimate them (e.g. reversible-jump), while jointly sampling OU parameters [56]. Such an approach was recently implanted in the R package bayou [57], and allows an inference of the location, magnitude, and number of adaptive shifts for univariate models. We hypothesize that the evolution of an aquatic lifestyle in pinnipeds will cause a selective regime shift at the basal node of pinnipeds which could be detected by these OU models.

Although OU-based methods are a powerful tool for testing alternative biological interpretations and ‘painting’ regime shifts onto branches of a phylogeny [54,57], they do not provide estimations of ancestral states nor of variable rates for individual branches. To overcome this empirical hurdle while avoiding increased model parameterization, [58] and [59] developed an approach (‘Independent Evolution’) that relies on similar assumptions as a multiple regime OU model, but requires fewer parameters. This approach assumes that population phenotypes are affected by the wandering adaptive peaks of adaptive surfaces (aligning with an Adaptive Peak model of evolution), and is therefore similar to an OU model with shifting locations in assuming that different regimes may occur at different locations in phylogenetic space. However, the formalization of IE assumptions differs considerably to the OU approaches, as the IE method utilizes a geometric approach that consists of two main steps: (1) quantifying an expected value for each internal node based only on phylogenetic and trait data, assuming a pure gradual mode of evolution; (2) quantifying the deviation of internal nodes to the expected value based on gradual evolution using a triangulation between the expected value and the two descendant values of the internal node in question. The triangulation between the gradual mode expectation of the ancestor and the observed descendants results in a rescaling of branches such that the barycentre among them provides the best fit for the data [58]; a procedure akin to Farris optimization [60,61]. The rate parameter is the distance between the barycentre of the triangulation procedure and the descendant value of the branch. This method therefore provides variable rate estimates for individual branches, as well as ancestral values for individual nodes. Under an adaptive radiation model, we would predict an increase in evolutionary rates on the branches at the base of Pinnipedia, relative to those higher up the pinniped phylogeny.

Measuring rates of evolution captures an important aspect of shape or trait evolution, but high rates of evolution may not translate simply into high diversity, or vice versa. For example, if taxa are constrained developmentally or ecologically to a particular range of shapes, they may show high rates of evolution and high amounts of convergence in form, but low overall morphological disparity [17,62,63]. In this scenario, analyses could accurately recover high rates of evolution but this would not show that the taxa of interest are repeatedly exploring the same range of morphospace rather than expanding into new morphologies. Alternatively, a clade could achieve high disparity through slow evolution if each shift moved into new regions of morphospace. Combining analyses of rates and morphological disparity thus allows for a more complete analysis of tempo and mode in the evolution of diversity. Under a model of diffusive evolution, we would predict that fissipeds would have greater disparity than fissipeds because fissipeds are the more taxonomically diverse and ancient clade.

## Methods

### Data set

This study included 151 specimens of adult carnivorans, including 64 fissiped and 87 pinniped specimens. These specimens constituted 34 fissiped species and 28 extant pinniped species (Table 1). Fissipeds are more taxonomically diverse than pinnipeds (241 and 36 species respectively), so fissipeds were relatively less densely sampled [46]. Specifically, we measured 14% of fissiped species, and 30% of fissiped genera. In contrast, 77% of pinniped species and all genera were included [46]. We were testing if the greater taxonomic diversity of fissipeds was reflected in a larger morphological disparity, so we sampled as broadly as possible within the group. Fissiped species were selected to encompass their full range of phylogenetic diversity, including representatives from every extant family and major ecological group. Although fossil fissipeds were unavailable for this study, previous studies suggest that most fossil fissipeds (except sabre-toothed cats) fall within the range of morphospace of extant clades [33]. The difference in sampling between fissipeds and pinnipeds may influence the results of variance-based disparity analyses as follows. Variance-based disparity measures may be overestimated in fissipeds relative to pinnipeds, because more dissimilar taxa were sampled for fissipeds, in order to cover their extant phylogenetic and ecological breadth without fully sampling their species diversity, than for pinnipeds, for which most extant and some fossil species were sampled. However, as our null hypothesis is that fissiped disparity should exceed pinniped disparity (due to their greater alpha diversity), this will not lead to a false rejection of the null hypothesis (type 1 error), but will make it more difficult to reject (type 2 error). Further, rarefaction will be used to statistically account for the differences in sampling (see below for details).

**Table 1:** **Species list, sampling and taxonomic assignment**

**Group**	**Family**	**Species**	**N**
**Feliformia**			
Fissiped	Eupleridae	*Cryptoprocta ferox*	1
Fissiped	Eupleridae	*Eupleres goudotii*	2
Fissiped	Eupleridae	*Fossa fossana*	2
Fissiped	Eupleridae	*Galidia elegans*	2
Fissiped	Felidae	*Acinonyx jubatus*	2
Fissiped	Felidae	*Felis bengalensis*	2
Fissiped	Felidae	*Felis vivverina*	2
Fissiped	Felidae	*Lynx rufus*	2
Fissiped	Herpestidae	*Cynictis penicillata*	1
Fissiped	Herpestidae	*Ichneumia albicauda*	1
Fissiped	Hyaenidae	*Crocuta crocuta*	2
Fissiped	Hyaenidae	*Proteles cristatus*	2
Fissiped	Nandinidae	*Nandinia binotata*	2
Fissiped	Viverridae	*Civettictis civetta*	2
Fissiped	Viverridae	*Genetta genetta*	2
Fissiped	Viverridae	*Paradoxurus hermaphroditus*	2
**Caniformia**			
Fissiped	Canidae	*Canis lupus*	2
Fissiped	Canidae	*Cerdocyon thous*	2
Fissiped	Canidae	*Otocyon megalotis*	2
Fissiped	Canidae	*Vulpes vulpes*	2
Fissiped	Mephitidae	*Mephitis mephitis*	2
Fissiped	Mustelidae	*Enhydra lutris*	2
Fissiped	Ailuridae	*Ailurus fulgens*	1
Fissiped	Mustelidae	*Gulo gulo*	1
Fissiped	Mustelidae	*Martes pennanti*	2
Fissiped	Mustelidae	*Meles meles*	2
Fissiped	Procyonidae	*Nasua nasua*	2
Fissiped	Procyonidae	*Potos flavus*	2
Fissiped	Procyonidae	*Procyon cancrivorous*	2
Fissiped	Procyonidae	*Procyon lotor*	2
Fissiped	Ursidae	*Ailuropoda melanoleuca*	2
Fissiped	Ursidae	*Melursus ursinus*	1
Fissiped	Ursidae	*Tremarctos ornatus*	2
Fissiped	Ursidae	*Ursus americanus*	2
Pinniped	Desmatophocidae	*†Allodesmus sp.*	1
Pinniped	Enaliarctinae	*†Enaliarctos emlongi*	1
Pinniped	Odobenidae	*Odobenus rosmarus*	3
Pinniped	Odobenidae	*†Pontolis magnus*	1
Pinniped	Otariidae	*Arctocephalus australis*	2
Pinniped	Otariidae	*Arctocephalus galapogoensis*	1
Pinniped	Otariidae	*Arctocephalus gazella*	5
Pinniped	Otariidae	*Arctocephalus philippi*	1
Pinniped	Otariidae	*Arctocephalus pussillus*	3
Pinniped	Otariidae	*Arctocephalus tropacalis*	2
Pinniped	Otariidae	*Callorhinus ursinus*	2
Pinniped	Otariidae	*Eumetopias jubatus*	3
Pinniped	Otariidae	*Neophoca cinerea*	1
Pinniped	Otariidae	*Otaria flavescens*	3
Pinniped	Otariidae	*Zalophus californianus*	1
Pinniped	Phocidae	*†Acrophoca longirostris*	1
Pinniped	Phocidae	*†Piscophoca pacifica*	1
Pinniped	Phocidae	*Cystophora cristata*	3
Pinniped	Phocidae	*Erignathus barbatus*	1
Pinniped	Phocidae	*Halichoerus grypus*	5
Pinniped	Phocidae	*Histriophoca fasciata*	7
Pinniped	Phocidae	*Hydrurga leptonyx*	2
Pinniped	Phocidae	*Leptonychotes weddelli*	3
Pinniped	Phocidae	*Lobodon carcinophagus*	4
Pinniped	Phocidae	*Mirounga leonina*	3
Pinniped	Phocidae	*Monachus monachus*	2
Pinniped	Phocidae	*Ommatophoca rossi*	2
Pinniped	Phocidae	*Pagophilus groenlandica*	1
Pinniped	Phocidae	*Phoca hispida*	4
Pinniped	Phocidae	*Phoca largha*	4
Pinniped	Phocidae	*Phoca vitulina*	2
Pinniped	Phocidae	*Pusa caspica*	4
Pinniped	Phocidae	*Pusa sibirica*	4

Species means were used in all analyses. Dagger indicates fossil taxa.

Several fossil pinnipeds were also available for study. Stem pinniped fossils were particularly important for the comparative analyses (disparity analyses were extant-only) because they provide information about the ancestral morphology of the group. These taxa may improve estimations of rates on the pinniped stem branches which spanned the terrestrial-aquatic transition. Cranial material of stem musteloids (sister taxa to pinnipeds) would have also been useful in this capacity, but were unavailable.

*Enaliarctos emlongi* (USNM 250345) is a stem pinniped from the Miocene (~20 mya) of California and represents an early radiation of enaliarctine pinnipeds. *Allodesmus sp.* (USNM 335445) is also from the Miocene of California but this specimen has not yet been carefully assessed in terms of its phylogenetic and taxonomic position. It is currently referred to the genus *Allodesmus*, which is a member of Desmatophocidae, an extinct pinniped family that has been variably related to either the Otarioidea or the Phocoidea [45,64-66]. *Pontolis magnus* (USNM 335567) is an extremely large fossil odobenid from the lower Pliocene Empire Formation of California and is thought to be closely related to the extinct Dusignathinae [67,68]. The two fossil phocids, *Acrophoca longirostris* (USNM 421632) and *Piscophoca pacifica* (USNM 360406) are both from the Pisco Formation of the Pliocene of Peru and are thought to be relatives of the monachine seals [69,70].

**Figure 2:**
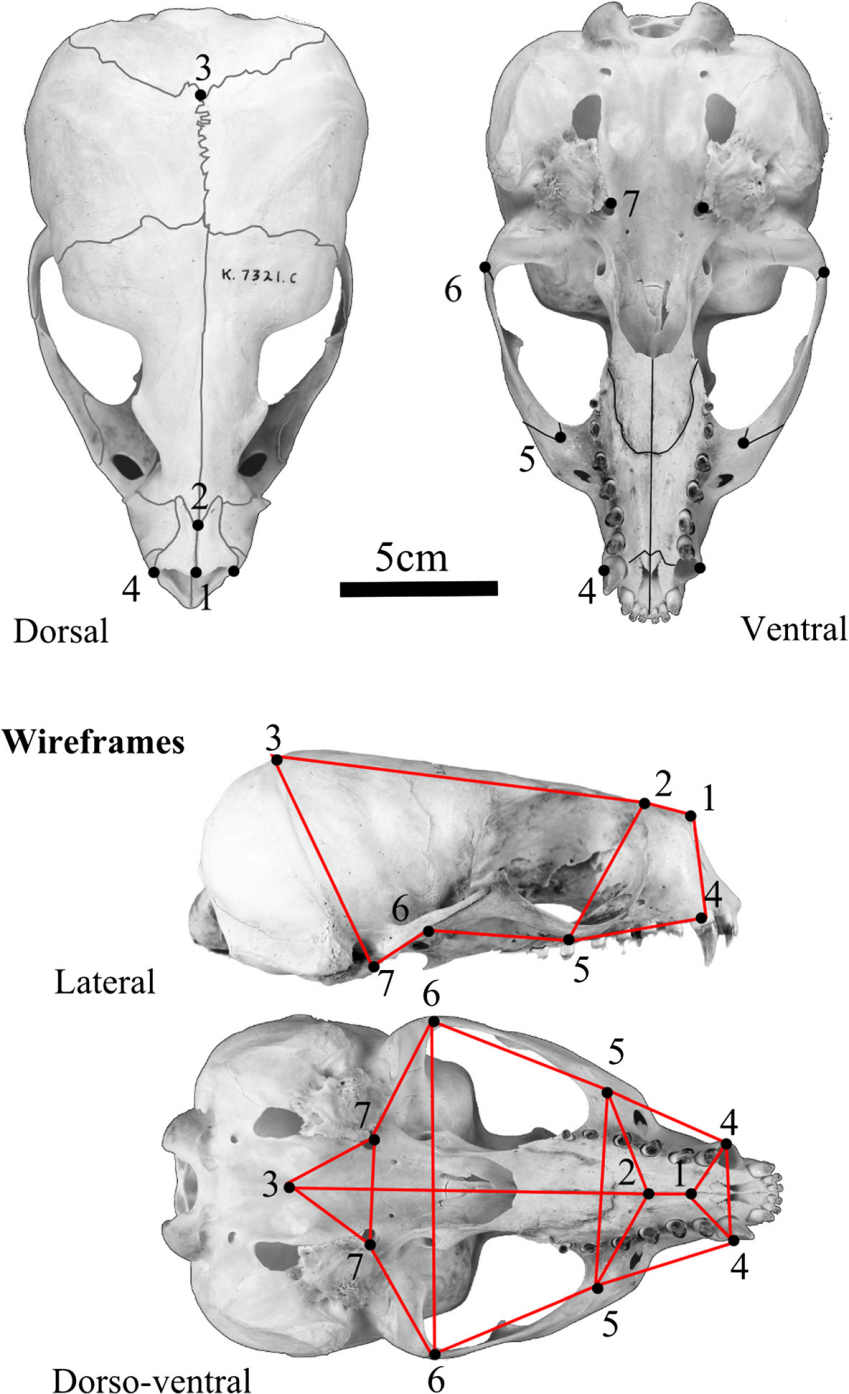
**11 landmarks used in data analysis, shown on a skull of** ***Arctocephalus gazella*** **.** Landmarks 4–7 were taken bilaterally. Landmark descriptions can be found in Table 2. Wireframe used to present shape variation from PCA shown in red.

Specimens were obtained from the University of Cambridge Zoology Museum, the Natural History Museum (London), the United States National Museum of Natural History (Washington, D.C.), the Field Museum of Natural History (Chicago), and the American Museum of Natural History (New York). Specific details on landmark collection, unification, and mirroring (to fill in missing data) are described in [71,72] and [31]. From the datasets detailed in those studies, a subset of 11 overlapping landmarks, observable and with clear homology in both fissipeds and pinnipeds, and identifiable in all the fossils, was selected. These cranial landmarks are shown in Figure 2 and described in Table 2, and the complete dataset is available at http://www.goswamilab.com. This number is reduced from the original datasets because of the need to capture equivalent landmarks on disparate skull shapes and incomplete material. However, the common landmarks still include information from most regions of the skull and provide much more information about skull proportions than would equivalent linear measures.

**Table 2:** **Description of landmarks taken**

**Number**	**Landmark**
1	Nasal midline
2	Nasal-Frontal midline suture
3	Parietal-Occipital midline suture
4	Canine labial*
5	Jugal-Maxilla posteroventral suture*
6	Jugal-squamosal posteroventral suture*
7	Auditory bulla anteromedial extreme*

*bilateral landmark were taken on both sides.

Two specimens of *Ailuropoda melanoleuca* were digitized by both observers to test inter-observer error. The average Procrustes distance between repeats (0.055) was smaller than the than the distance between different specimens of *A. melanoleuca* (0.081), and much less than the average Procrustes distance between fissiped specimens and the mean (0.114).

### PCA

Three-dimensional landmark data were analyzed using the software MorphoJ [73]. Specimens were brought into the same shape space (removing all non-shape elements) via translation, rotation and scaling by Procrustes superimposition. Further, the Procrustes co-ordinates were symmetrized and the symmetric component of variation used in subsequent analyses. Previous work indicates that shape variation of the carnivoran cranium is limited enough to apply the tangent-space approximation [74]. Species averages were then calculated from specimen data so that each tip on the phylogenetic tree is represented by a single shape point [3]. Though most species were represented by multiple individuals sampling ranged from 1 to 7 individuals. Quantifying intraspecific variation is not critical here because we are measuring large-scale cross-taxonomic morphological variation. Any fossil specimens with high asymmetry were removed prior to analysis, as unusually high asymmetry may reflect distortion that occurred during or after fossilization. Principal components analysis was used to reduce the dataset to orthogonal principal components (PCs) for subsequent analysis. These PC data were used in disparity and rate analyses, as opposed to Procrustes co-ordinate scores, to provide more convenient and interpretable graphing of the evolutionary morphospace through time, and to overcome computing limits on variable numbers in some analyses. However, both data sets provide the same shape data when all the PCs are included.

### Comparisons of disparity

The variation in shape within a group can be expressed in many different ways, and the disparity measure chosen may impact interpretation of the data. Hence, multiple measures of disparity should be used to fully understand the patterns of variation observed [3,75]. Range-based methods identify the maximal morphospace occupation of each group, and therefore are more sensitive to outliers and sample size bias. However they do inform about the maximum differences in shape within a group. Here, the sum of ranges across all PCs was used as a measure of range. Variance-based disparity metrics also include information about the spread of species within the morphospace, but are much more robust to sampling bias [3]. For example, a tightly clustered group with a few divergent species might have the same range as a widely dispersed group, but would have a lower variance. The mean multivariate distance to the group centroid was used as the variance-based disparity metric. Estimates of the total variance of each group should be less sensitive to uneven sampling than the convex hull area [75,76].

These metrics were calculated using the MDA package in Matlab [76,77] for fissipeds and extant pinnipeds. Fossil pinnipeds were excluded from this analysis to provide a fairer comparison to fissipeds, for which fossil specimens were unavailable. Disparity analyses were carried out using all principal component scores in order to take into account all of the variation in the dataset. To compute standard deviations and confidence intervals on the disparity measures, a bootstrap procedure was used. Specimens were re-sampled randomly with replacement 1000 times, and the mean (disparity value) and deviation calculated. In addition, to take into account the uneven sampling between fissipeds and pinnipeds, rarefaction was used. The pinniped sample was rarefied to a sample-size of five using bootstrapping. This represents an equivalent species-sampling to that of fissipeds.

### Evolutionary rates

The phylogenetic relationships and branch lengths used for estimating evolutionary rate were taken from [40]. Fossil species were then added based on positions indicated by [41] and [70], with branch lengths equivalent to their earliest appearance in the fossil record (Figure 1). Due to controversy over the position of the walrus, and the potentially large impact of this key node to our interpretations, rate analyses were run using both the Otarioidea and Phocomorpha hypotheses.

A summary of comparative analyses run in this study can be found in Table 3. To investigate the relative likelihood of alternative evolutionary scenarios of shifts in cranial morphology in the carnivoran phylogeny, we used the ouch *R*-package [78] to fit Ornstein-Uhlenbeck models to multivariate data. To investigate the likelihood of an evolutionary scenario in which pinnipeds indicate a higher rate of change at their basal branch, we tested four alternative models. The first model is a neutral Brownian motion model, which assumes that evolutionary change and selection follow a random walk, and therefore does not align with adaptive radiation assumptions. The second model considers a single optimum along the entire phylogeny, consistent with a shared selective regime across carnivorans. The third model considers two separate optima, for terrestrial and aquatic carnivorans. This hypothesizes regime shifts both at the base of Pinnipedia and on the branch leading to the otter. Finally, the fourth model considers an adaptive shift at the base of pinnipedia, in concert with the reconstructed position of the terrestrial-aquatic transition. The analysis was run on the top 9 PCs which together account for 95.2% of variation (multivariate hypothesis test). The analyses included the top 9 PCs rather than all PCs due to the extensive time required to run the models.

**Table 3:** **Rate and comparative analyses summary**

**Analysis**	**Data**	**Model**	**Implementation**	**Presented in**
Multivariate IE	All PCs	Independent Evolution	evomap, Euclidean distances	Figure 7, Additional file 10
Multivariate hypothesis test	PC1-9	Ornstein-Uhlenbeck	ouch	Table 5
Univariate IE	PC1-4	Independent Evolution	evomap	Figure 6, Additional files 1, 2, 3, 4, 5, 6, 7, 8 and 9
Univariate OU	PC1-4	Ornstein-Uhlenbeck	bayou	Additional files 11, 12, 13, 14, 15, 16, 17 and 18

To describe morphological changes along individual branches of the phylogeny we employed the R packages evomap [79] and bayou [57]. The IE method (available in evomap) provides both ancestral states and variable rates, allowing for a detailed description of how morphospace changed through evolutionary time along individual branches of the phylogeny. These changes can be visualized into an evolutionary morphospace that captures changes through time by taking snapshots of morphospace along intervals of time. This approach is different from the more widely used ‘phylo-morphospace’ approach [17] in that the evo-morphospace approach consists of visualizing the evolutionary changes in the morphospace through time, rather than the projection of a phylogeny into a morphospace. The evolutionary morphospace approach, available in evomap [79], thus fully captures evolutionary trends by displaying how morphospace is inferred to have changed over time through phylogenetic space. Rates and reconstructed node values were calculated for all PCs individually using evomap, of which the top 4 are presented (univariate IE analysis). From the node values, the Euclidean distance between ancestor–descendant pairs across all PCs was calculated, providing an estimate of total evolutionary change along each branch of the tree (multivariate IE analysis).

The method implemented by bayou allows the inference of the location, magnitude and number of adaptive shifts within a multiple-optima OU framework, hereby providing a detailed inference of selective shifts in the phylogeny (Univariate OU analysis). We applied this analysis to the top 4 PC’s as an independent validity check of results obtained by the IE method.

## Results

### Cranial shape of fissiped and pinniped carnivorans

**Figure 3:**
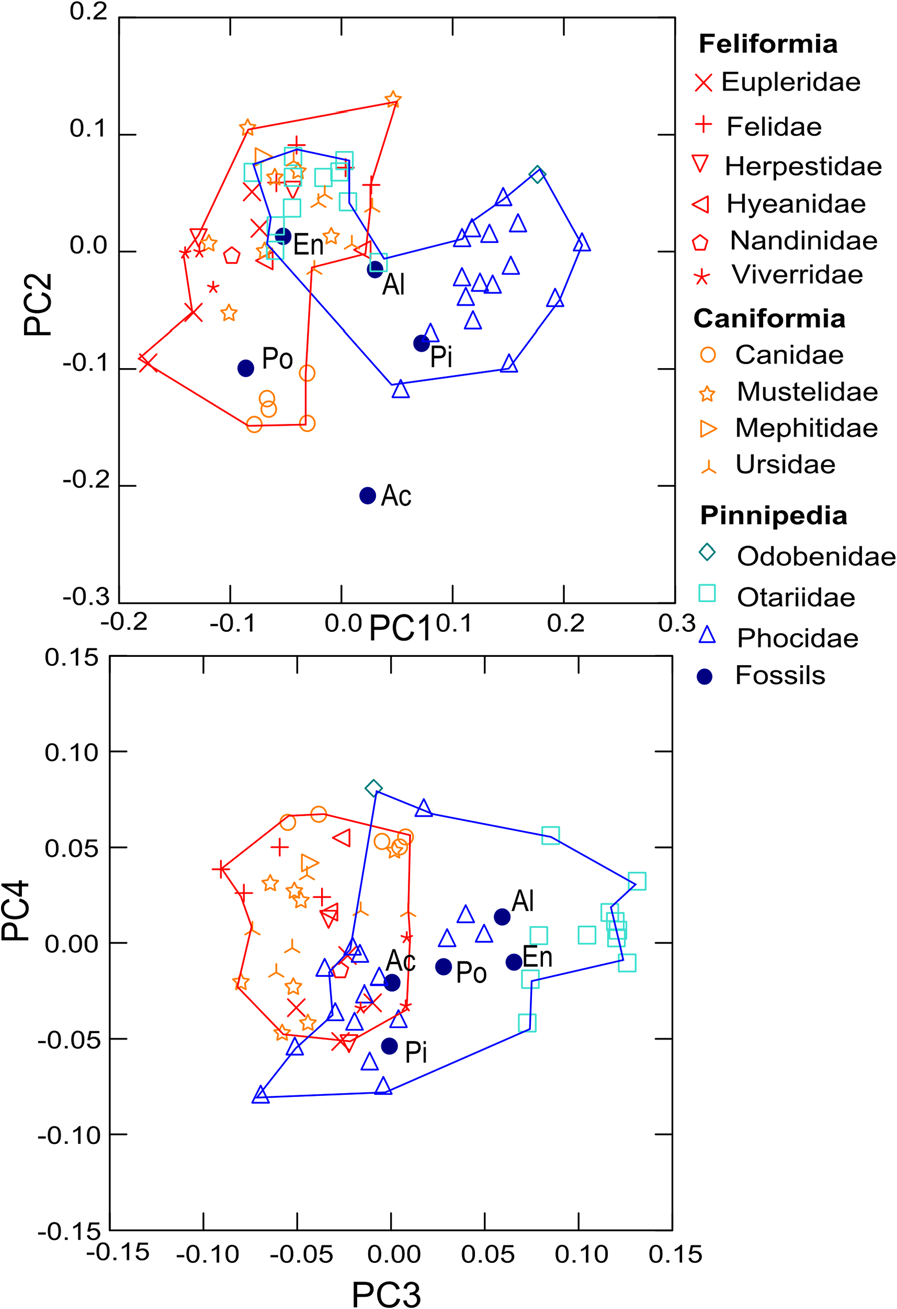
**Scatterplots showing variation on PC1 - PC4. These axes represent 39.76%, 20.36%, 14.92% and 6.14% of variance.** Based on species means. Fossil pinnipeds are as follows: En, *Enaliarctos emlongi*; Al, *Allodesmus* sp*.*; Po, *Pontolis magnus*; Pi, *Piscophoca pacifica*; Ac, *Acrophoca longirostris.* Polygons connect fissipeds (red) and extant pinnipeds (blue) and reflect groupings used in the disparity analyses. Extremal shapes are shown in Figure 4.

**Figure 4:**
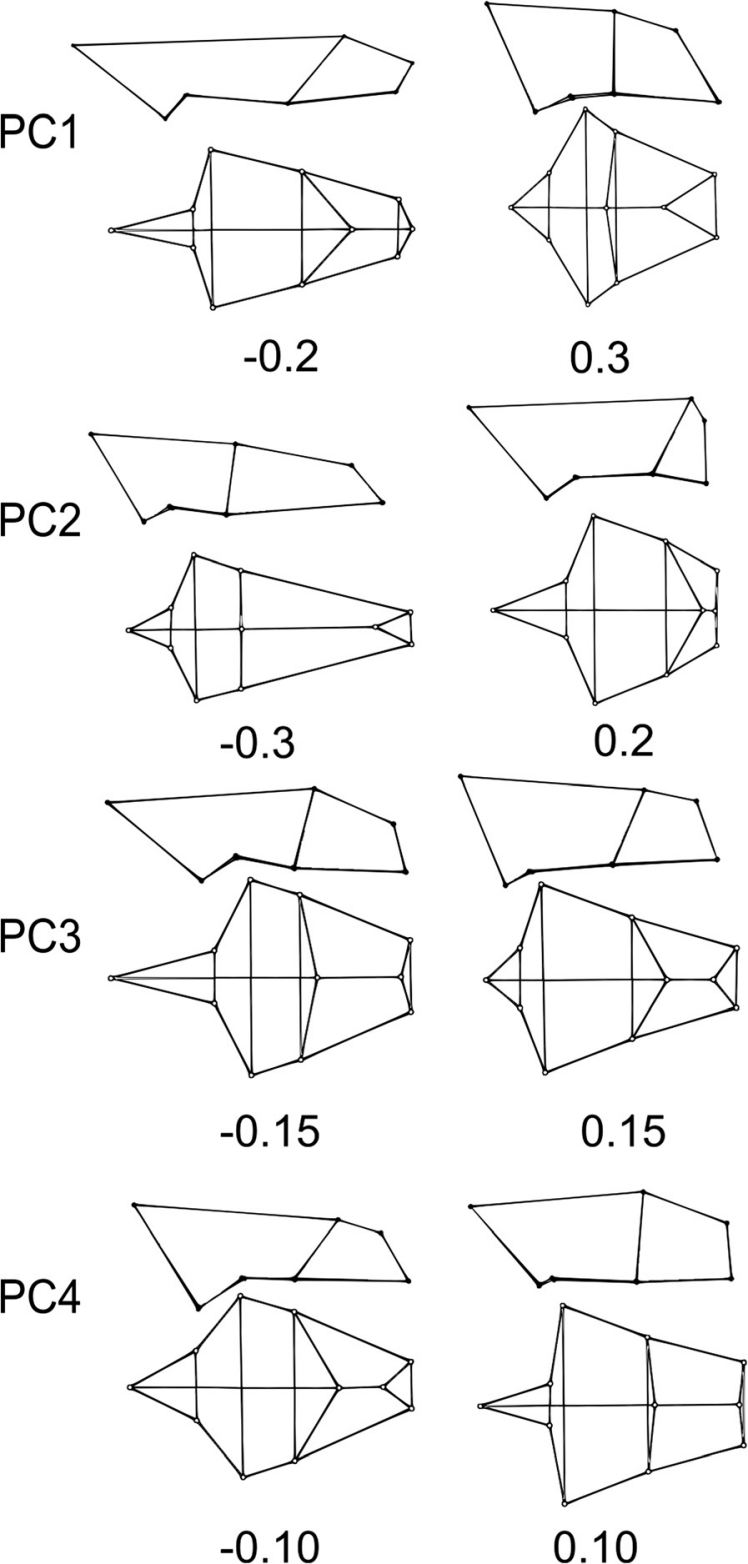
**Wireframes showing shape variation on PC1-PC4 in lateral and dorsal views.** Anterior is to the right of the image. Landmarks the wireframe was based on are shown in Figure 2.

**Figure 5:**
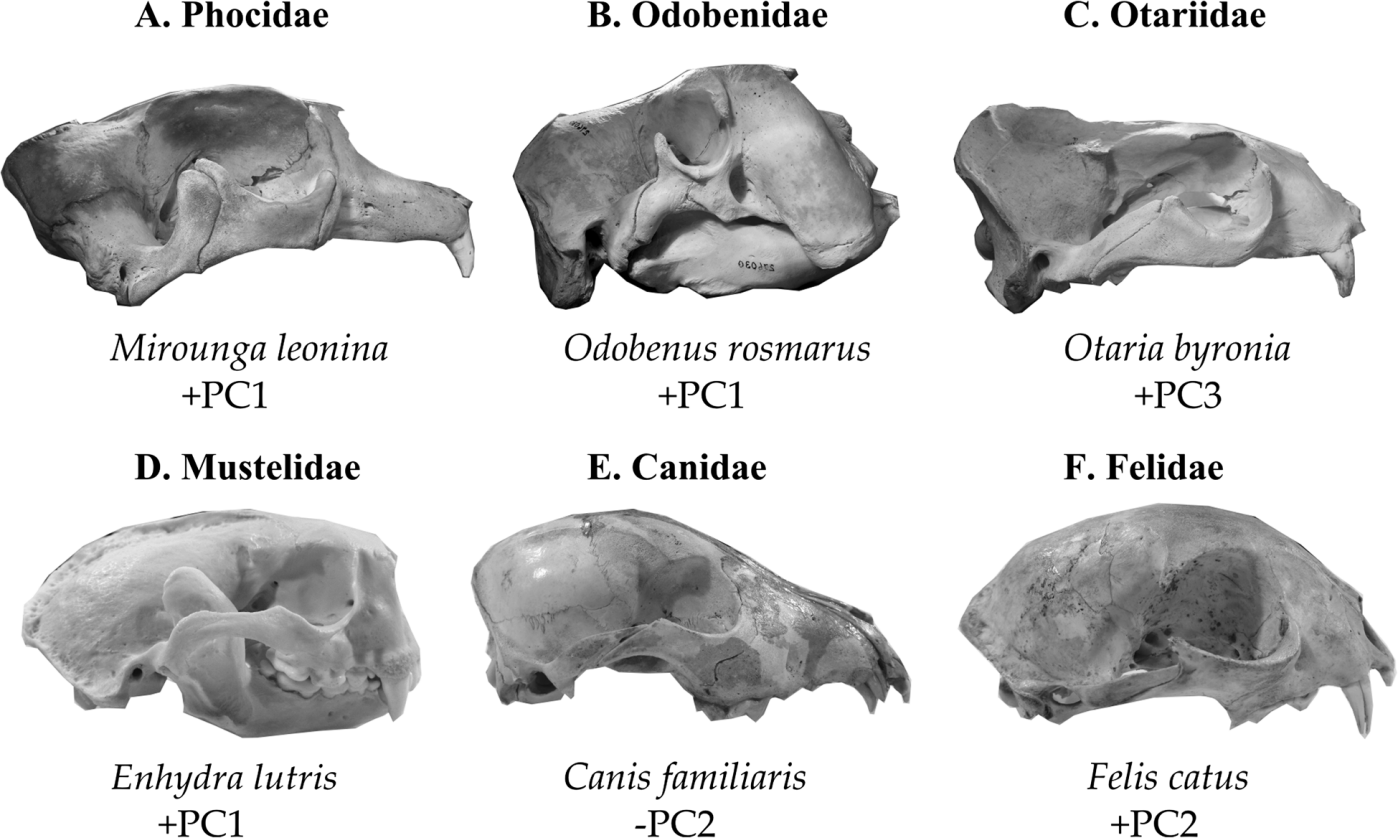
**Morphological variation in carnivoran skulls. A-C**: representatives of the three families of pinnipeds. **D-F**: Examples of shape variation within fissipeds. Note the enlarged nasal opening typical of positive PC1 scores, found in phocids, odobenids, and the fissiped otter. Dog and cat represent dolichocephalic and brachiocephalic extremes respectively, reflected by PC2 score.

Figure 3 depicts the first and second principal components which account for 60.12% of the total variance. Illustration of shape variation is provided by the wireframes in Figure 4 and the comparison plate of skull photographs in Figure 5. PC1 distinguished phocid and odobenid pinnipeds from otariids and fissipeds. Phocids, with high scores on PC1, displayed large nasal openings, dorso-ventrally tall and mediolaterally wide crania. This is in comparison to the relatively dorsoventrally flat and mediolaterally narrow terrestrial skull that has more anteriorly positioned nasal bones. The aquatic mustelid *Enhydra lutris* (sea otter, superior-most orange star) fell close to otariid space and in the most positive position on PC1 among fissipeds, although some ursids (orange trefoil) also displayed relatively high PC1 scores. The stem pinniped *E. emlongi* and the desmatophocid *Allodesmus sp.* were similar to the otariids on this axis, whereas the fossil phocids *P. pacifica* and *A. longirostris* fell within or close to extant phocids.

PC2 reflected snout elongation across Carnivora. Short-faced (brachycephalic) species, such as *Enhydra,* displayed positive scores on PC2, whereas long-snouted (dolichocephalic) species, like most canids, had negative scores. The fossil taxa *A. longirostris* (the long-necked seal), and *Pontolis magnus* (a stem odobenid), as well as the extant leopard seal, *Hydrurga leptonyx*, had crania that were very dolichocephalic.

Figure 3 also illustrates PC3 and 4, which combined represent 21.06% of the variance in the analyses. PC3 distinguished otariids from other pinniped and fissiped groups. Otariids differed in the more anterior placed parietal-occipital suture relative to the bullae, longer ventral portion of the jugal, and more anteriorly place nasofrontal relative to the jugal-maxillary suture. Both *E. emlongi* and *Allodesmus sp.* were near to, but just outside of, otariid morphospace on this axis. PC4 represents around 6% of variation and did not distinguish at all fissipeds from pinnipeds, which overlap fully on this axis.

### Disparity analyses

Results of disparity analyses of fissiped and pinniped carnivorans can be found in Table 4. For pinnipeds, results are shown both for the full number of specimens, and for a sample which has been rarefied down to the equivalent fissiped sampling-level (proportional to their taxonomic diversity) using bootstrapping (five specimens). The sum of ranges, a range-based disparity metric, is more sensitive to sample size. When all pinnipeds are included, pinniped disparity was larger, but the pinniped confidence interval overlapped with the fissiped value, indicating that there was not a significant difference in disparity between the two groups. However, when pinnipeds are bootstrapped the range-based disparity falls drastically, such that it is below the lower confidence limit of fissipeds. The mean distance to the centroid, a variance-based disparity metric, was much more robust to sampling changes. In this case, both original- and rarefied-pinniped disparity was above the 95% confidence interval for fissipeds, indicating that pinnipeds had higher variance, which was robust to sampling.

**Table 4:** **Results of disparity analyses**

	**N**	**Sum of ranges**	**Lower 95% CI**	**Upper 95% CI**	**Mean dist. centroid**	**Lower 95% CI**	**Upper 95% CI**
Fissipeds	34	1.511	1.399	1.603	0.107	0.096	0.120
Pinnipeds (all)	28	1.659	1.472	1.786	0.134	0.120	0.147
Pinnipeds (rarefied)	5	1.052	0.758	1.311	0.134	0.120	0.145

CI: confidence interval. Ninety five percent confidence interval is from 1000 bootstraps.

### Evolutionary rates

**Figure 6:**
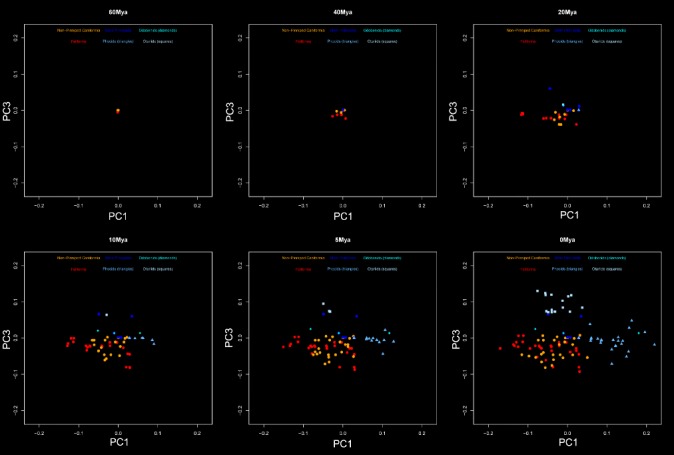
**Evolutionary morphospace showing the reconstructed evolution of Carnivora on PC1 and PC3 through time.** Based on IE analysis. Phylogeny and colors as shown in Figure 1. Points at the zero time point represent both nodes and tip values.

**Figure 7:**
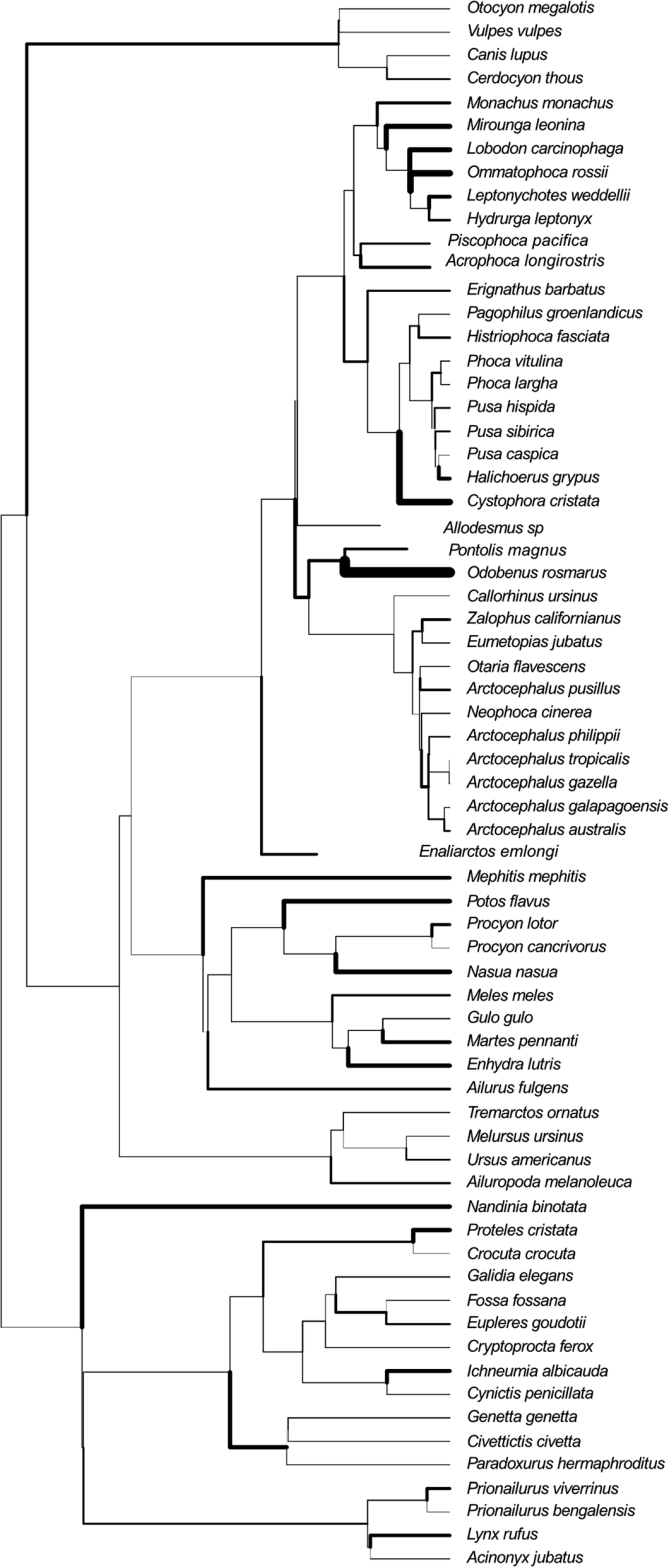
**Multivariate IE analysis results showing shape change on each branch.** Multivariate evolutionary distances are calculated from node estimates based on IE analysis. Thicker branches represent greater morphological change on that branch.

Evolutionary rates on the carnivoran tree were assessed using both Independent Evolution (evomap) and Ornstein-Uhlenbeck (bayou and ouch) models. Results for the IE model can be found in Figures 6, 7, and Additional files 1, 2, 3, 4, 5, 6, 7, 8, 9 and 10, bayou results in Additional files 11, 12, 13, 14, 15, 16, 17 and 18, and ouch results in Table 5.

**Table 5:** **Results of multivariate hypothesis test in ouch**

	**BM**	**One regime**	**Terrestrial-aquatic**	**Pinniped radiation**
Phocomorpha	**−2155.8**	−860.4	−913.3	−1187.3
Otarioidea	**−2155.8**	−828.2	−1192.5	−1157.4

Akaike information criterion (AIC) values for the four hypotheses tested in ouch. Based on data from the top 9 PCs representing 95.2% of variation in the sample. BM, Brownian motion model; One regime, single optimum for all Carnivora; Terrestrial-aquatic, one regime for terrestrial carnivorans, another for pinnipeds plus the otter; Pinniped radiation, one regime for fissipeds, another for pinnipeds. Bold shows best AIC values for the BM hypothesis in all cases.

The AIC values for the four hypotheses tested using the multivariate ouch model-fitting approach are shown in Table 5. The BM model is supported for both Phocomorpha and Otarioidea. This suggests that there is no single selective regime influencing all carnivorans, nor is there a drastic shift in selective regime at the base of pinnipeds, but instead support a randomly varying adaptive peak.

The IE and bayou univariate rate analyses based on the Otarioidea hypothesis paint a similar picture of trait evolution for the first 4 PCs (Additional files 1, 11, 12, 13 and 14). On PC1, the highest evolutionary rates within pinnipeds are found in crown phocids and on the *O. rosmarus* branch, and similarly bayou finds regime shifts at the base of Phocidae and on the *O. rosmarus* branch. On PC2, which represents snout length, the canids have high evolutionary rates and high likelihood of a regime shift, associated with their dolichocephalic morphology, as does the long-snouted fossil pinniped *A. longirostris.* PC3 is associated with high evolutionary rates on, and a regime shift at, the base of the Otariidae. These patterns support the ouch results as they show that selection is acting in different morphological directions for the pinniped families (phocids and odobenids versus otariids), and cannot be united by a single selection regime at the base of the group. The results based on Phocomorpha are extremely similar to those based on Otarioidea, with the exception that the bayou model places an additional morphological shift on the stem Phocomorpha branch. Results for the Phocomorpha analyses can be found in Additional files 2, 15, 16, 17 and 18. In short, neither the evomap method nor the bayou method provides support for a rate or regime shift at the basal branch of pinnipeds.

Univariate IE analyses of individual PC scores can be mapped to show the morphological changes through time along individual branches of the phylogeny, producing an evolutionary morphospace graph. Figure 6 shows the evolutionary morphospace of PC1 against PC3, the two axes linked with fissiped-pinniped variation, for the Otarioidea hypotheses. Similar plots for PC1 and PC2, can be found in Additional file 3. Videos showing the evolutionary morphospace through time are presented in Additional files 4 and 5. Figure 7 shows that the fissipeds (orange and red) continue to evolve in the lower left region of the morphospace (negative PC1 and PC3) throughout the 60 million years. However, the appearance of crown pinnipeds marks the invasion of new areas of the morphospace. Crown phocids and otariids evolve quickly into the positive regions of PC1 and PC3 respectively, with many points showing similar trajectories. Results for the Phocomorpha analysis are essentially the same, and can be found in Additional files 6, 7, 8 and 9.

The total evolutionary change along each branch of the phylogeny was estimated with a multivariate IE analysis using all individual PCs. Results of this multivariate analysis are shown in Figure 7 for the Otarioidea hypothesis. The amount of shape change on each branch of the tree is indicated by its line thickness. Results of the same analysis using the Phocomorpha hypothesis are similar, and so are included in Additional file 10. These results do not indicate a marked morphological shift at the basal branch of the pinnipeds, but match the results of the previous analyses in suggesting evolutionary shifts higher in the pinniped tree. Within pinnipeds, the highest evolutionary shifts are found on branches leading to the walrus (*Odobenus rosmarus*), the hooded seal (*Cystophora cristata*), and within crown monachine seals. Branches connecting crown group pinnipeds, which reflect the original radiation of pinnipeds, generally indicate lower amount of shape change (Figure 7, Additional file 10). In the fissipeds, the highest amount of shape change tends to be at terminal branches, including those leading to *Enhydra lutra, Mephitis mephitis, Proteles cristata, Nasua nasua* and *Potos flavus,* but are also quite high on the stem branch of the Canidae.

## Discussion

During the ~43ma of their evolution, crown-group carnivorans have diversified into many ecological niches and geographical areas [33,80-82]. However, invasion of the aquatic environment is arguably the largest ecological transition in carnivoran evolution [44]. This study has demonstrated that although pinniped carnivorans have distinctive cranial morphology to fissipeds, and relatively large disparity for their taxonomic age; this high disparity was not achieved by rapid evolution at the base of Pinnipedia, as would be predicted under an adaptive radiation model. Instead, pinnipeds evolved at low-moderate rates at the base of the group, followed by later spikes of increased rate on terminal branches.

### Fossil pinnipeds

Principal component analysis on cranial shape demonstrated that pinniped and fissiped carnivorans occupy distinctive regions of morphospace. In particular, phocids and odobenids are primarily distinguished from fissipeds on PC1, representing a dorsoventrally tall, mediolaterally wide skull, with high and posteriorly-placed nasal bones increasing the size of the nasal opening associated with dorsally-placed nares. Similarly, otariids are distinguished from fissipeds on PC3 by their anteriorly placed nasofrontal suture and long jugals. The inclusion of fossil pinnipeds in this study provides promising preliminary results concerning the extinct diversity and evolutionary history in this group. The stem pinniped, *E. emlongi,* shared similar cranial morphology to otariids, confirming previous qualitative work [48,83,84]. This also demonstrates that, despite the fact that *E. emlongi* is phylogenetically intermediate between fissipeds and pinnipeds, in terms of its skull morphology it can be fully distinguished from extant fissipeds on PC3, supporting inferences from post-crania that enaliarctines were likely fully adapted for an aquatic lifestyle [83,85].

*Allodesmus* sp. is intermediate between phocids and otariids, reflecting its phylogenetic position as a member of the Desmatophocidae, an enigmatic clade that is likely the sister group to one of those two families [45,64]. In contrast, the stem odobenid *P.magnus* lies far from the extant walrus in morphospace, with a relatively dolichocephalic morphology compared to the extant walrus. This suggests that the vast taxonomic diversity of extinct, stem odobenids may have encompassed a much larger amount of morphological disparity than other pinniped or fissiped clades and did not progress linearly from an *Enaliarctos-*type ancestral morphology to that of modern walruses [41]. Inclusion of more fossil walruses into future analyses could clarify evolutionary patterns in this diverse group.

One of the most unusual fossil pinnipeds, *A. longirostris,* the long-necked seal, has a score on PC1 similar to that of other phocids; however, it is more dolichocephalic than any living phocid. Dolichocephaly is very common in fissipeds and is thought to be related to the variable expression of the Runx2 gene [74,86]. This illustrates that some aspects of phenotypic variation are important in both fissipeds and pinnipeds, and is reflected by strong overlap between the two groups on PC2, representing dolichocephalic-brachiocephalic morphologies. In fact, *A. longirostris* is more dolichocephalic than any extant fissiped, including canids, raising questions about the functional implications of this divergent skull morphology. This species is known from the Pliocene of Peru and although it’s feeding ecology is not well understood, it has features consistent with the generalized pierce feeding typical of the group, but with interdigitate tooth cusps that have been linked with filter feeding in extant groups [87].

### Fissiped and pinniped cranial disparity

Disparity analyses revealed that fissipeds do not have greater morphological disparity than extant pinnipeds, despite their greater taxonomic diversity and earlier divergence age. In this study, pinnipeds were sampled much more densely than fissipeds, which can lead to discrepancies in the measurement of disparity. In particular, fissiped specimens were selected broadly from across the group, to encompass their full range of diversity. However, this sampling scheme is likely to results in an inflated variance-based disparity, because taxa selected were more taxonomically diverse. Therefore, it is even more surprising that pinnipeds had greater variance than fissipeds. To further account for these sampling differences, the pinniped sample was rarefied down to a similar sampling level. While between-group patterns remained the same for variance-based disparity, range-based disparity was much lower in the rarefied sample. This reflects that range-based disparity is very sensitive to sample size, whereas variance-based metrics are not.

These data suggest that despite their younger taxonomic age and constituting relatively fewer species, pinnipeds have more variable skull morphology than the sampled fissipeds. This supports some previous data which showed fissipeds have relatively low cranial disparity compared to other mammalian groups [25], but is surprising given the large diversity of dietary specialization in the group [81]. Pinnipeds, on the other hand, are generally opportunistic feeders, but include a number of cranial specializations for prey capture and sexual display or combat [31]. These range from tusks and suction feeding specializations in the walrus to the bizarre inflatable nasal balloon in the hooded seal, and demonstrate the remarkable plasticity of cranial form in this group.

Heterogeneous disparity patterns such as these have been found in other carnivoran groups [74]. Domesticated dogs (a single species) have cranial disparity equivalent to the rest of the order, likely related to strong selection pressures during artificial selection. Those data showed that changing selection regimes (for features favored by dog breeders) have resulted in evolution of novel skull shapes, outside those found in wild Carnivora [74]. Despite this, modularity and integration patterns, which reflect the mechanisms generating natural variation, are not altered. Pinnipeds have also attained increased cranial diversity and evolved novel morphology outside the range of the other carnivorans. Hence both artificial and natural selection environments can influence cranial disparity in the Carnivora.

### Adaptive radiation models in Pinnipedia

We did not find support for an increase in the rate of cranial evolution in stem pinniped lineages, nor a systematic shift in selective regime associated with the terrestrial-aquatic transition. Additionally, there was only a small amount of multivariate shape change on pinniped basal branches. All these results point to more gradual evolution across the terrestrial-aquatic transition. In contrast, the highest rates of evolution and strongest selective regime shifts were found within the crown group. The adaptive radiation model predicts that evolutionary rates should be high at the base of the radiation, then gradually slow as the niche fills. Therefore, these results do not provide support for an adaptive radiation model in the evolution of pinnipeds based on the current sample. This result was recovered using both Phocomorpha and Otarioidea arrangements, demonstrating that it is robust to the phylogenetic hypothesis used.

This result is in agreement with some other large-scale comparative studies which have shown that adaptive radiations may be quite rare [7,88]. In particular, very few groups were found which matched an early burst, followed by stasis, pattern. Those authors found that even though patterns tended to match constrained evolution, groups evolved too slowly for stabilizing selection [7]. Instead they suggest either oscillating selection or developmental constraints in shaping long-term evolutionary patterns. Similarly, [39] found similar rates of body size evolution in fissipeds and pinnipeds, suggesting that a large body size range in pinnipeds was achieved without an increase in evolutionary rate. Interestingly, angular measurements of the carnassials in carnivore evolution do display adaptive radiation-type patterns of evolution, establishing strong between-clade differences early in Carnivora [11,89,90]. However, the strong functional links between carnassial morphology and prey acquisition in fissiped carnivores may make this example a stronger candidate for an adaptive radiation model.

Despite the fact that evolutionary rates were low at the base of the pinniped radiation, increases in evolutionary rate were noted at other places within Pinnipedia. In particular, there were increased evolutionary rates in phocid terminal branches and the walrus branch. This matched the regime shifts detected by the OU model in phocids and *Odobenus* on PC1, indicating repeated selection and convergence toward a positive PC1 optimum. This suggests multiple instances of rapid evolution toward a tall cranium, with a highly expanded nasal opening and recessed nasal bones. This supports previous work based on similar data, which hypothesized convergent evolution of recessed nasals associated with sexual displays and sediment feeding in phocids [31]. For example, *M. leonina* and *C. cristata* have distinctive cranial morphology related to sexual dimorphism specializations, and both have relatively high rates of evolution [31]. Similarly, *O. rosmarus* is highly specialized for suction feeding, and has high estimated evolutionary rates, though fossil walruses were poorly sampled here. This suggests specialization for marine ecologies may be driving increased morphological rates in phocids, and that they may show relatively high plasticity with regard this type of morphological variation.

The bayou analysis, based on the Ornstein-Uhlenbeck model, and the evomap analysis, based on the independent evolution model, both suggest that the regime shifts for pinniped families were associated with different selective optima (positive PC1 vs PC3). This indicates selective divergence of the crown groups, and does not support a single unifying selective regime for the group. Phocids, odobenids and otariids differ in their ecology in several ways. For example, phocids inhabit higher latitudes, spend more time in water, use different swimming mechanisms and have more diverse dietary and reproductive strategies than otariids [44,85,91]. Therefore, there may have been bursts of evolution within crown Pinnipedia associated with exploiting new ecological opportunities within the aquatic realm.

As well as extrinsic factors, such as environment, it is also important to consider intrinsic factors [88]. One explanation for the high pinniped disparity is that evolution of an aquatic lifestyle released certain constraints acting on the terrestrial carnivoran skull. For example, mastication produces high loadings in the maxillary dentition which are transmitted through the rest of the skull [92-96]. Pinnipeds, and some other aquatic groups (e.g., cetaceans), do not masticate their food [44,97]. In concert with the loss of mastication, pinnipeds also evolved a shortened tooth row, homodont dentition and lost the typical mammalian tooth structure [34,87,98-101]. Instead of masticating with posterior teeth, pinniped behaviors (such as pierce feeding or male-male combat) involve biting with the anterior dentition. Loss of posterior tooth biting may therefore have changed the types of loadings experienced on the cranium as a whole and may have allowed pinnipeds to evolve new and disparate skull forms not accessible to fissipeds. The relaxation of functional constraints is one potential hypothesis for pinniped skull diversity which warrants future exploration. This hypothesis could be further investigated using functional analysis (e.g., FEA) of biting in the pinniped and fissiped cranium.

This theory is in line with the idea of an ‘escape from the terrestrial adaptive zone’. The concept of a terrestrial ‘adaptive zone’, or constrained area of morphospace associated with function, was originally suggested by [32] for the pinniped ankle. In order to examine the idea of a terrestrial adaptive zone in the cranium, we must first establish convergence of fissipeds within a confined region of morphospace and second demonstrate that morphological variation in the cranium is functionally related to their environment [32].

On the first point, there is strong overlap of feliform and fissiped caniform morphology on the first four PCs, suggesting convergence between species in both clades. Within the Caniformia, there is some overlap between mustelids and ursids, though canids have distinctive dolichocephalic cranial shapes. On the second point, there is ample evidence that skull morphology is strongly influenced by functional pressures. The morphology of the fissiped cranium is influenced by diet [28,33,81,102-106], sensory evolution [107] and thermoregulatory demands [108]. The pinniped skull is adapted for marine functions, such as aquatic prey acquisition, and polygynous reproductive strategies in island colonies [31,87,109]. Relative to their terrestrial ancestors, pinnipeds have specialized feeding systems [87,100,110-112], thermoregulation [113,114], sub-aquatic vision and hearing [115-119], diving [120,121] and intra-specific displays [31,122,123]. Therefore, we suggest that, similarly to the ankle, pinniped skull morphology may have diverged out of a constrained region of morphospace occupied by fissipeds by diffusive evolution, followed by stronger selection in different directions within crown pinniped families.

## Conclusion

The evolution of aquatic habits in pinniped carnivorans presents an ideal case study of the effect of ecological transitions on evolutionary processes because both terrestrial and aquatic groups are well represented in modern faunas. This study suggests that pinnipeds are more diverse in cranial morphology than are fissipeds, despite their more recent evolutionary origin. However, this increased disparity was not achieved through higher rates of evolution, or selective regime shifts, associated with the terrestrial-aquatic transition. Instead pinniped evolution is characterized initially by low to moderate evolutionary rates. Later in pinniped evolution, there were several bursts of more rapid evolution, associated with shifts in selection regime toward divergent optima, which may reflect ecological diversification within the aquatic realm. Thus we demonstrate that very high evolutionary rates in stem lineages are not necessary to produce morphological diversity. Instead ecological diversification within the aquatic realm, and perhaps the release of terrestrial constraints, seem to be important in driving pinniped cranial disparity.

## Additional files

Additional file 1:
**UnivariateIE_Otarioidea.pdf, figure, PC1-4 from IE analysis.** Thickness of branch is proportional to rate. Green, positive direction; red, negative direction. Additional file 2:
**UnivariateIE_Phocomorpha.pdf, figure, PC1-4 from IE analysis.** Thickness of branch is proportional to rate. Green, positive direction; red, negative direction. Additional file 3:
**IE_Evomorphospace_PC1PC2_otarioidea.pdf, figure, Evolutionary morphospace for PC1 vs PC2 for otarioidea.**
Additional file 4:
**Evomorphospace_timelapse_PC1PC3_otarioidea.mov, movie, Evolutionary morphospace movie for PC1 vs PC3 for otarioidea.**
Additional file 5:
**Evomorphospace_timelapse_PC1PC2_otarioidea.mov, movie, Evolutionary morphospace movie for PC1 vs PC2 for otarioidea.**
Additional file 6:
**IE_Evomorphospace_PC1PC2_phocomorpha.pdf, figure, Evolutionary morphospace for PC1 vs PC2 for phocomorpha.**
Additional file 7:
**IE_Evomorphospace_PC1PC3_phocomorpha.pdf, figure, Evolutionary morphospace for PC1 vs PC3 for phocomorpha.**
Additional file 8:
**Evomorphospace_timelapse_PC1PC2_phocomorpha.mov, movie, Evolutionary morphospace movie for PC1 vs PC2 for phocomorpha.**
Additional file 9:
**Evomorphospace_timelapse_PC1PC3_phocomorpha.mov, movie, Evolutionary morphospace movie for PC1 vs PC3 for phocomorpha.**
Additional file 10:
**MultivariateIE_phocomorpha.pdf, figure, Multivariate distances calculated from nodes from IE model.** Thickness of branches represents multivariate distance between ancestor–descendant. Additional file 11:
**Bayou_PC1_otarioidea.pdf, figure, Results of bayou analysis on PC1 for otarioidea.** Circles at the node represent the likelihood value of a shift occurring at that node. Nodes with a likelihood value of 0.2 or greater were mapped with a regime shift, represented by a change in color, on the subsequent branches. Additional file 12:
**Bayou_PC2_otarioidea.pdf, figure, Results of bayou analysis on PC2 for otarioidea.** Circles at the node represent the likelihood value of a shift occurring at that node. Nodes with a likelihood value of 0.2 or greater were mapped with a regime shift, represented by a change in color, on the subsequent branches. Additional file 13:
**Bayou_PC3_otarioidea.pdf, figure, Results of bayou analysis on PC3 for otarioidea.** Circles at the node represent the likelihood value of a shift occurring at that node. Nodes with a likelihood value of 0.2 or greater were mapped with a regime shift, represented by a change in color, on the subsequent branches. Additional file 14:
**Bayou_PC4_otarioidea.pdf, figure, Results of bayou analysis on PC4 for otarioidea.** Circles at the node represent the likelihood value of a shift occurring at that node. Nodes with a likelihood value of 0.2 or greater were mapped with a regime shift, represented by a change in color, on the subsequent branches. Additional file 15:
**Bayou_PC1_phocomorpha.pdf, figure, Results of bayou analysis on PC1 for phocomorpha.** Circles at the node represent the likelihood value of a shift occurring at that node. Nodes with a likelihood value of 0.2 or greater were mapped with a regime shift, represented by a change in color, on the subsequent branches. Additional file 16:
**Bayou_PC2_phocomorpha.pdf, figure, Results of bayou analysis on PC2 for phocomorpha.** Circles at the node represent the likelihood value of a shift occurring at that node. Nodes with a likelihood value of 0.2 or greater were mapped with a regime shift, represented by a change in color, on the subsequent branches. Additional file 17:
**Bayou_PC3_phocomorpha.pdf, figure, Results of bayou analysis on PC3 for phocomorpha.** Circles at the node represent the likelihood value of a shift occurring at that node. Nodes with a likelihood value of 0.2 or greater were mapped with a regime shift, represented by a change in color, on the subsequent branches. Additional file 18:
**Bayou_PC4_phocomorpha.pdf, figure, Results of bayou analysis on PC4 for phocomorpha.** Circles at the node represent the likelihood value of a shift occurring at that node. Nodes with a likelihood value of 0.2 or greater were mapped with a regime shift, represented by a change in color, on the subsequent branches.

## Abbreviations

PCA: Principal components analysis
PC: Principal components
USNM: United states national museum
IE: Independent evolution model
OU: Ornstein-Uhlenbeck model
