# Supplementary figures and images for: Impact of the terrestrial-aquatic transition on disparity and rates of evolution in the carnivoran skull
Source: BMC Evol Biol. 2015 Feb 4;15(1):8. doi: 10.1186/s12862-015-0285-5 (PMC4328284; doi:10.1186/s12862-015-0285-5)

PC1

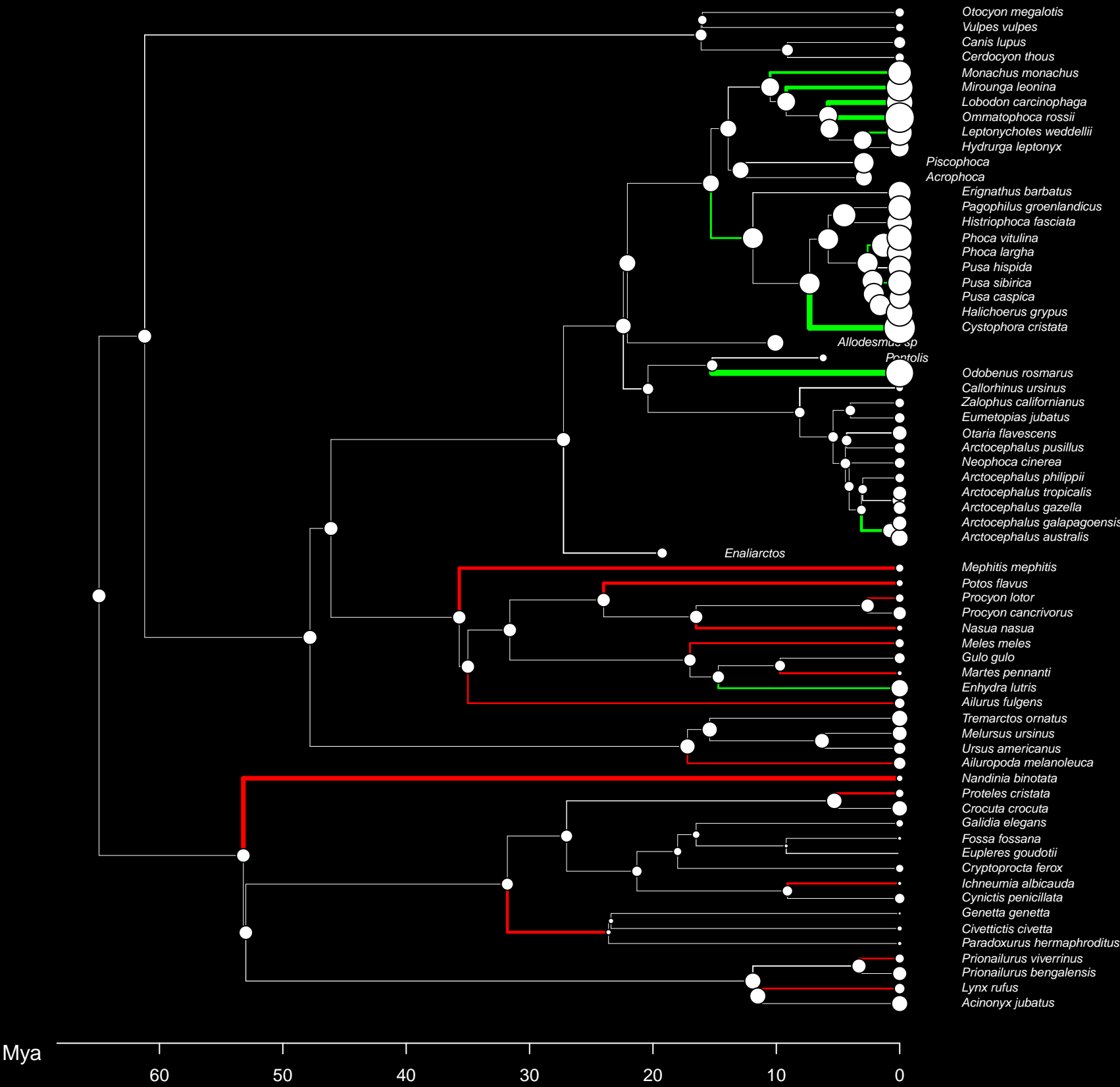

PC2

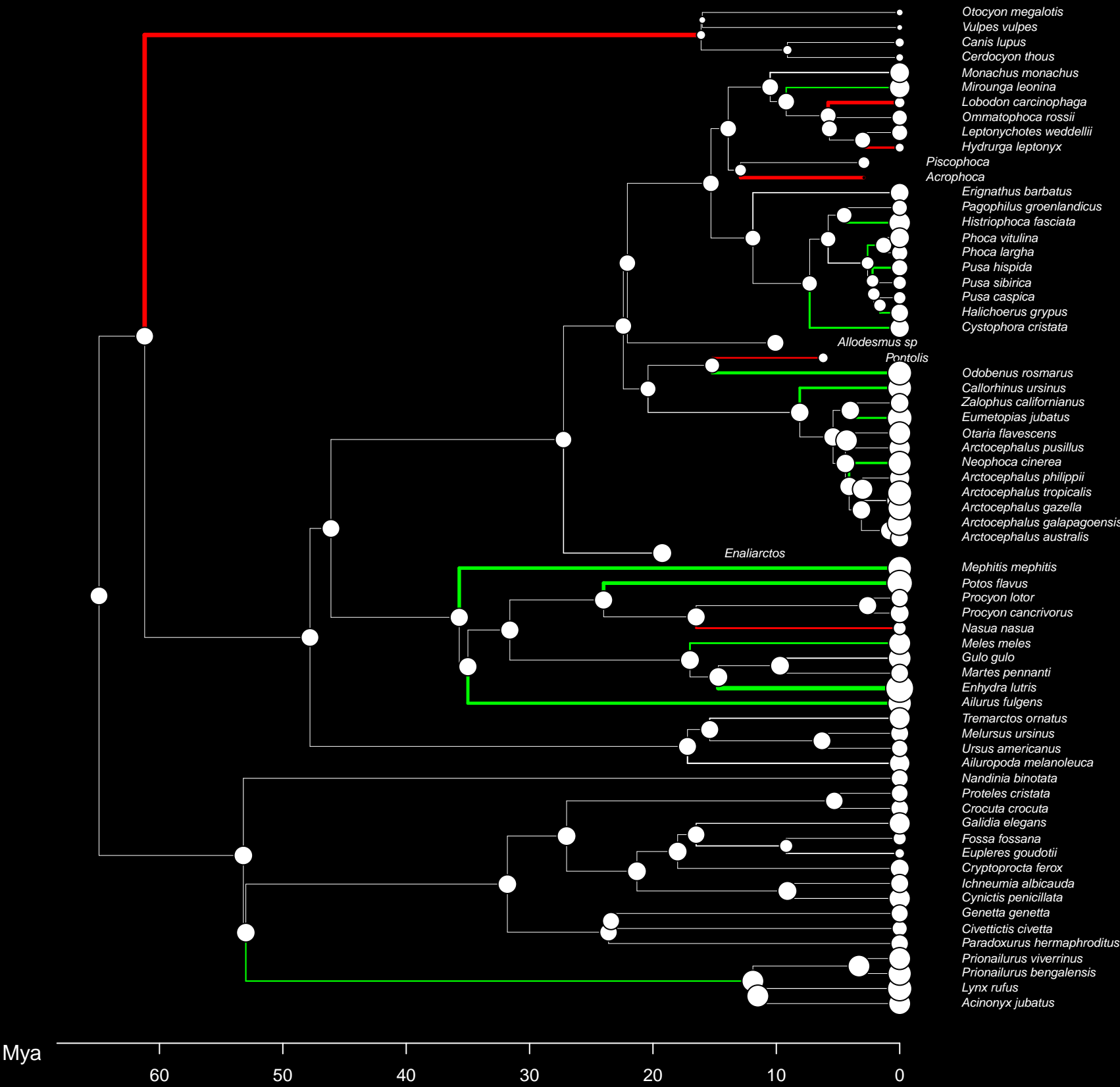

PC3

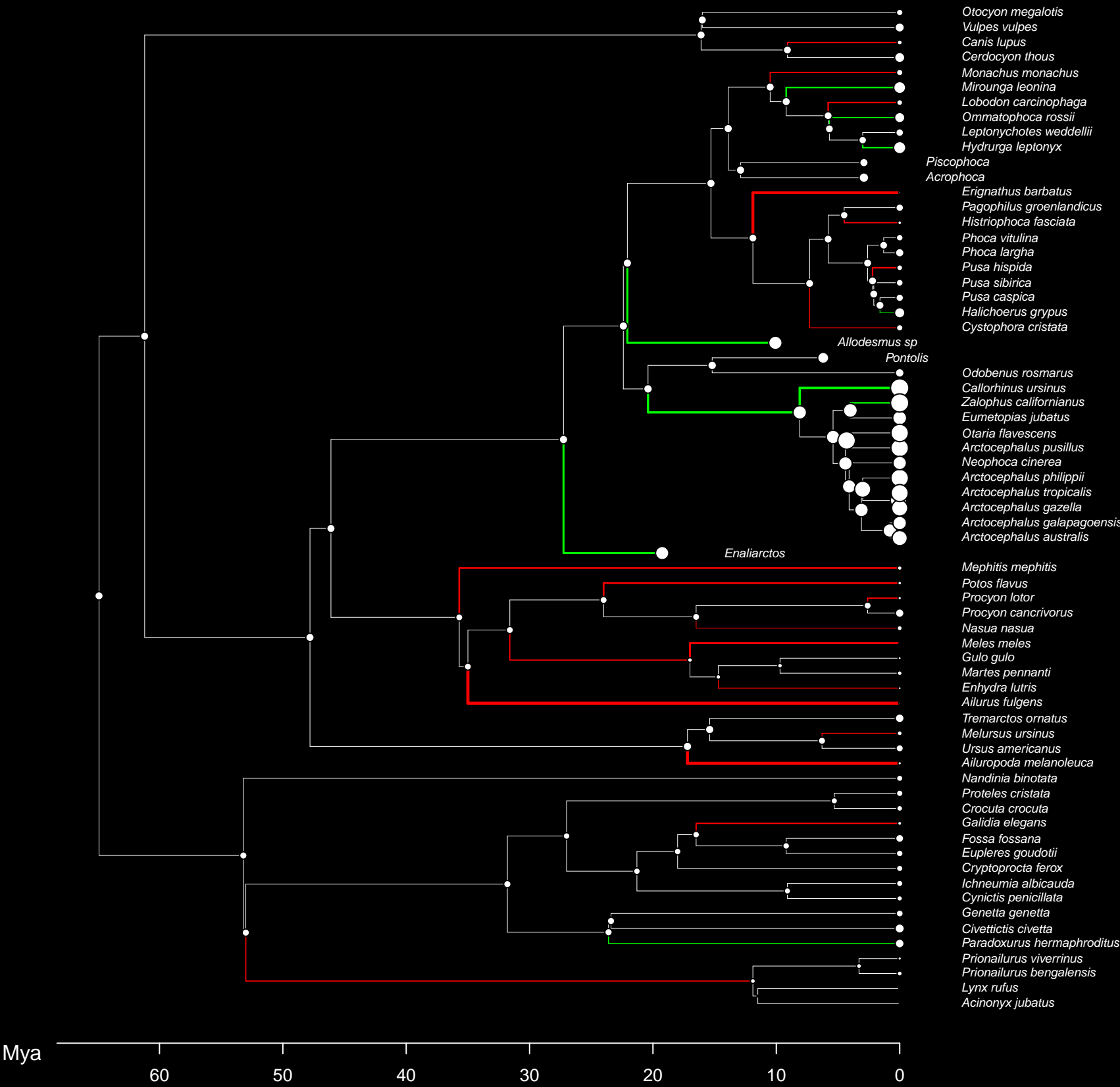

PC4

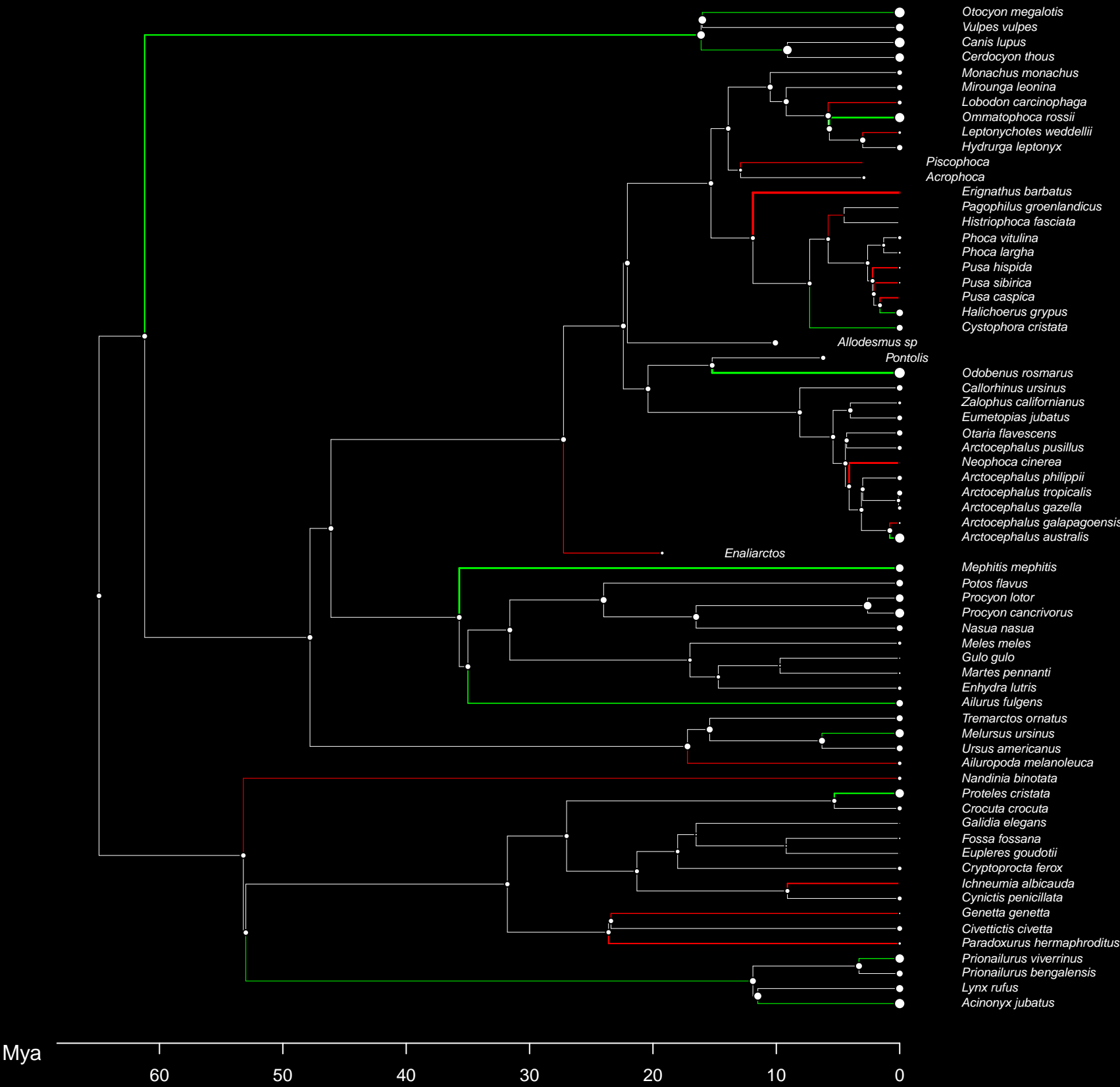

Supplement: Additional file 1: — UnivariateIE_Otarioidea.pdf, figure, PC1-4 from IE analysis. Thickness of branch is proportional to rate. Green, positive direction; red, negative direction. [file 12862_2015_285_MOESM1_ESM.pdf]

PC1

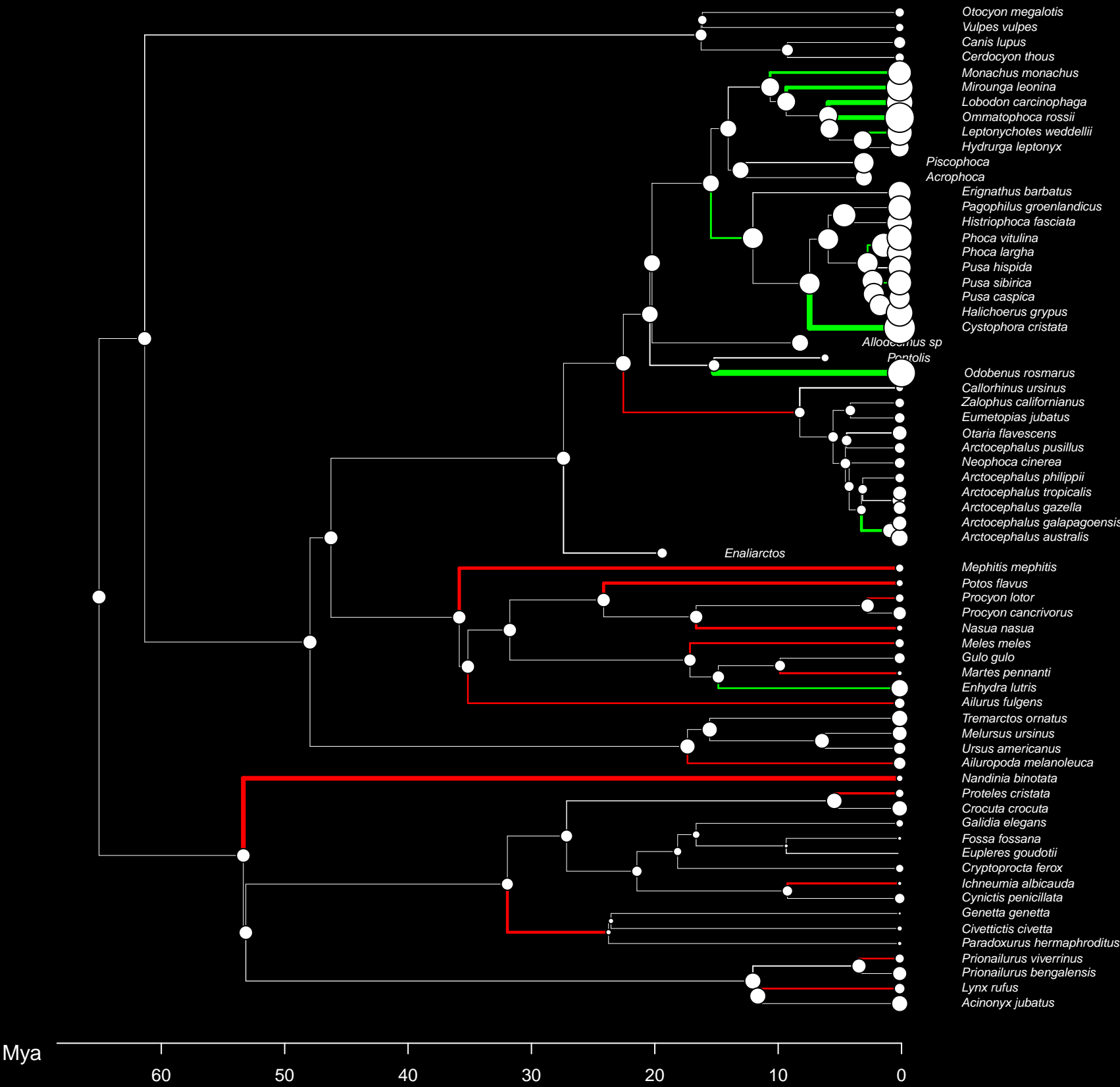

PC2

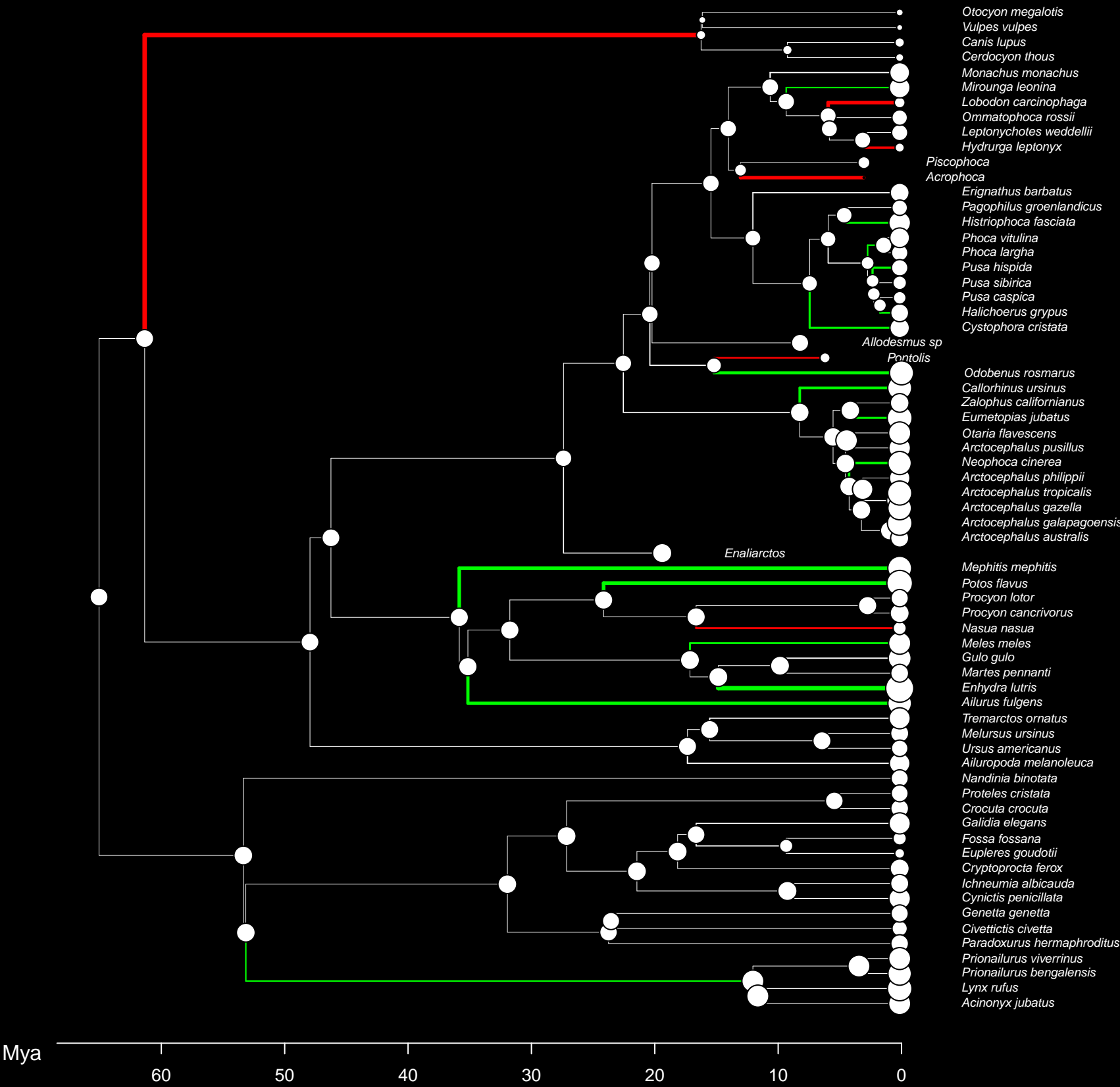

PC3

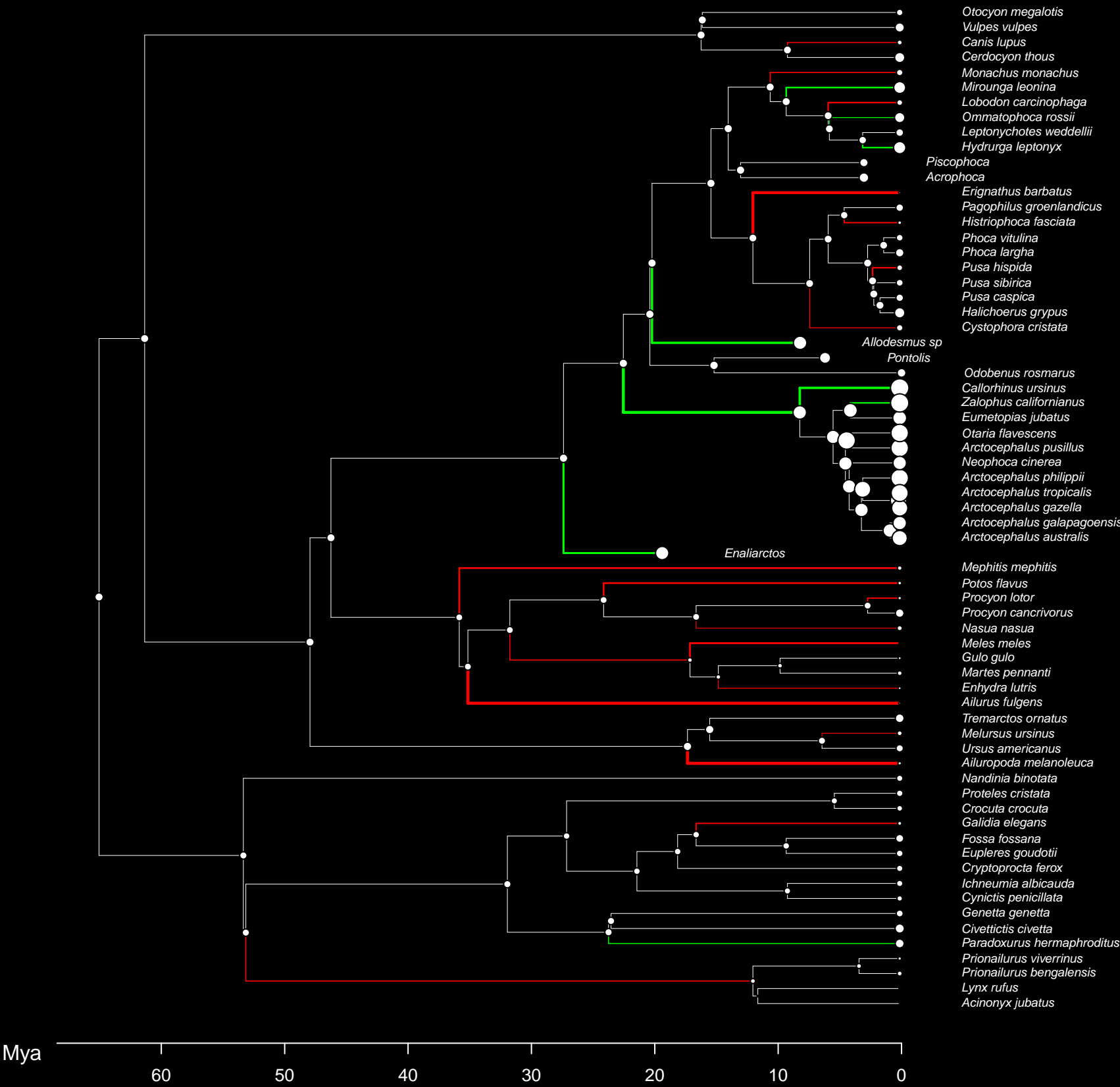

PC4

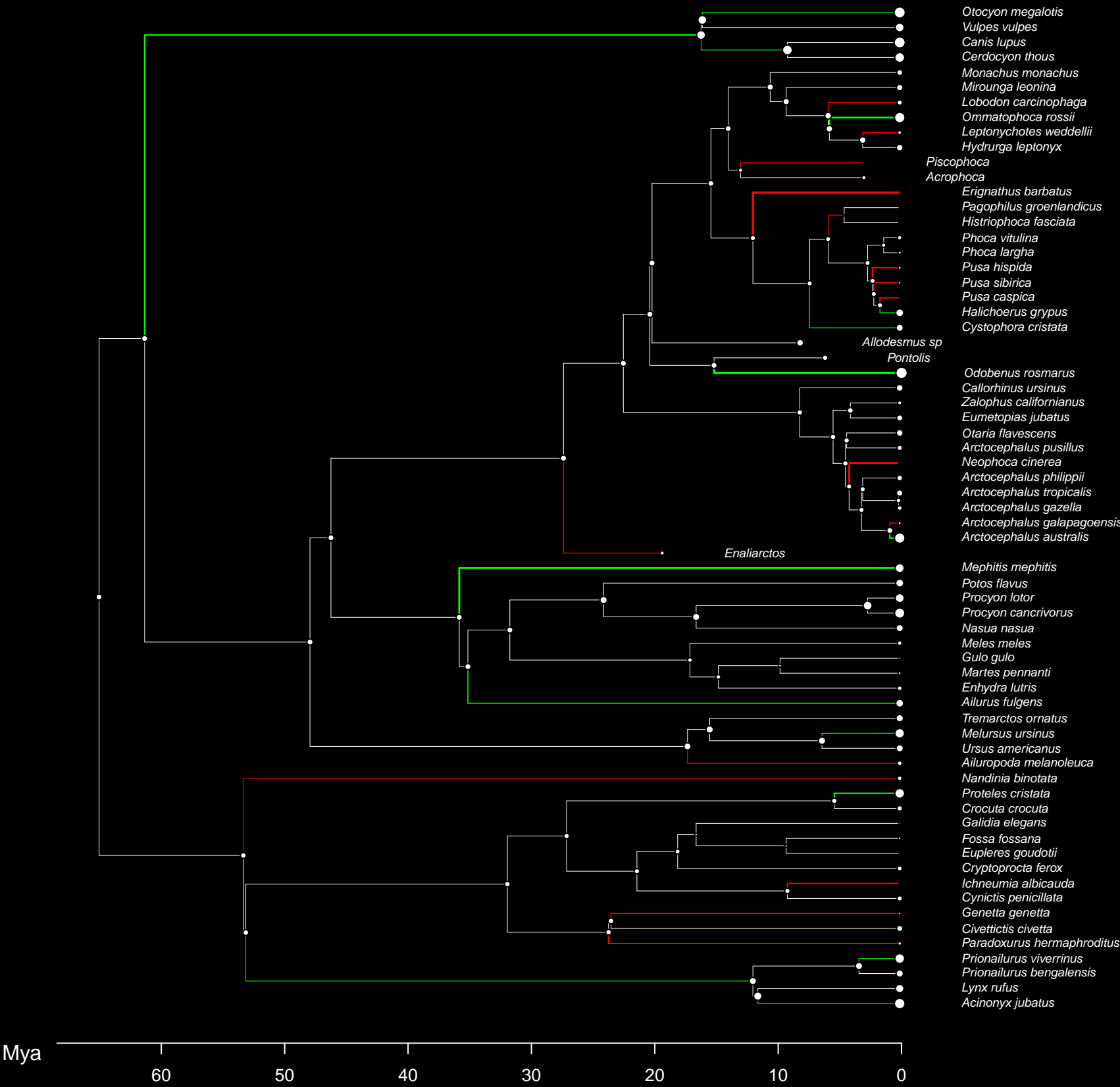

Supplement: Additional file 2: — UnivariateIE_Phocomorpha.pdf, figure, PC1-4 from IE analysis. Thickness of branch is proportional to rate. Green, positive direction; red, negative direction. [file 12862_2015_285_MOESM2_ESM.pdf]

60Mya

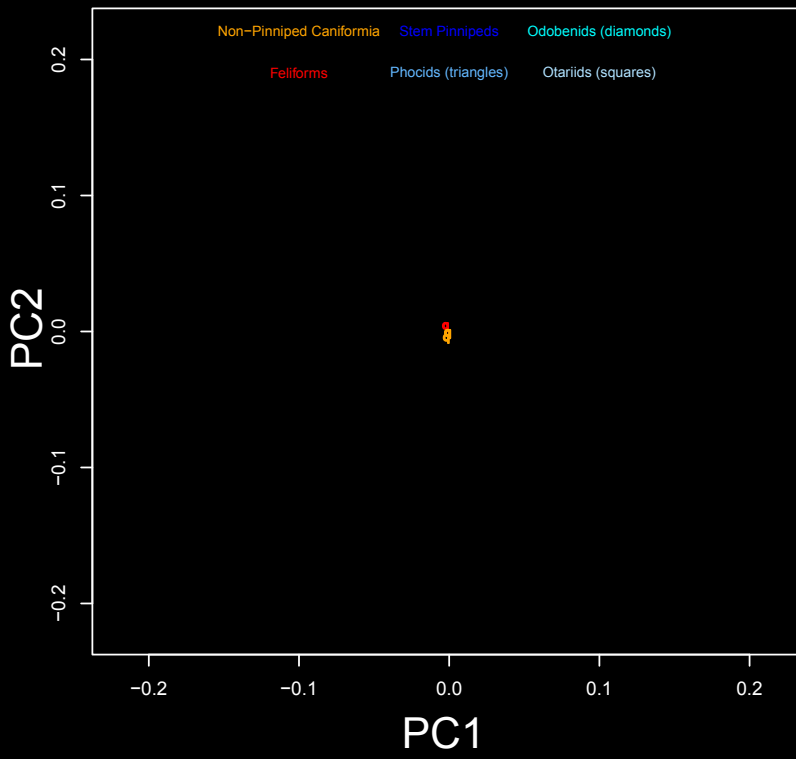

40Mya

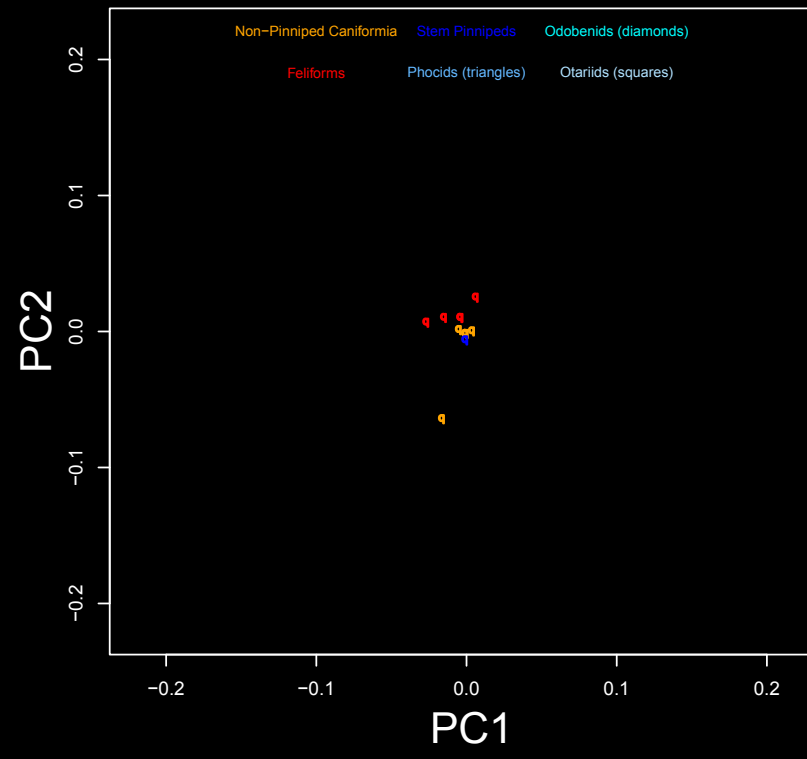

20Mya

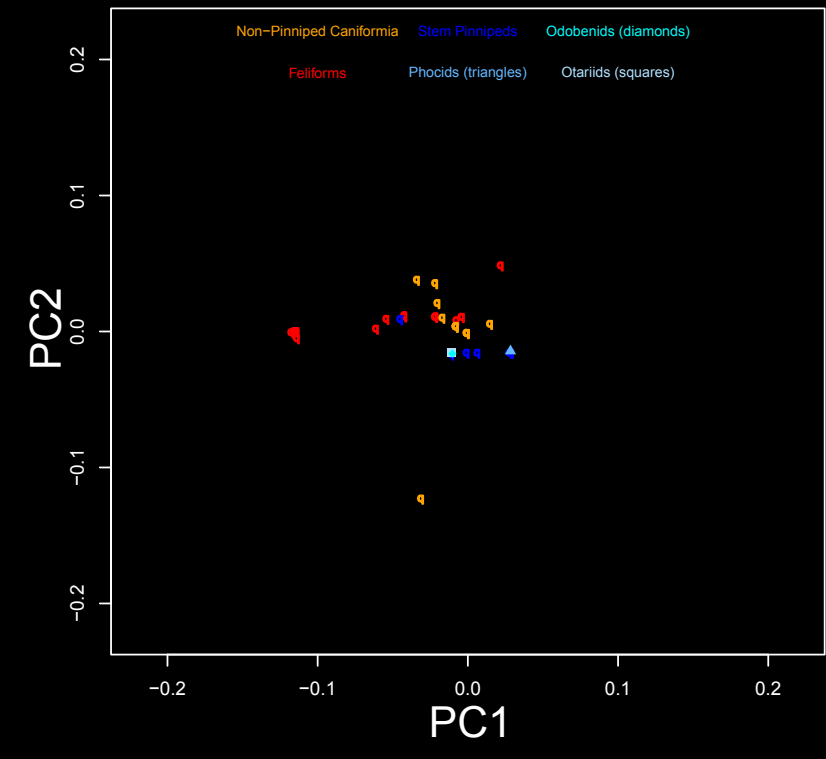

10Mya

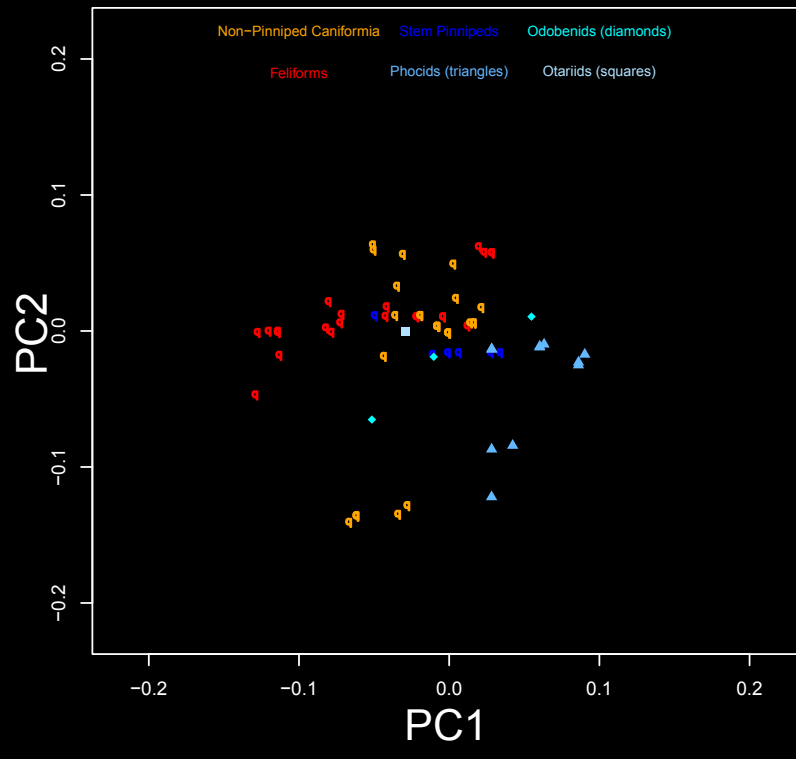

5Mya

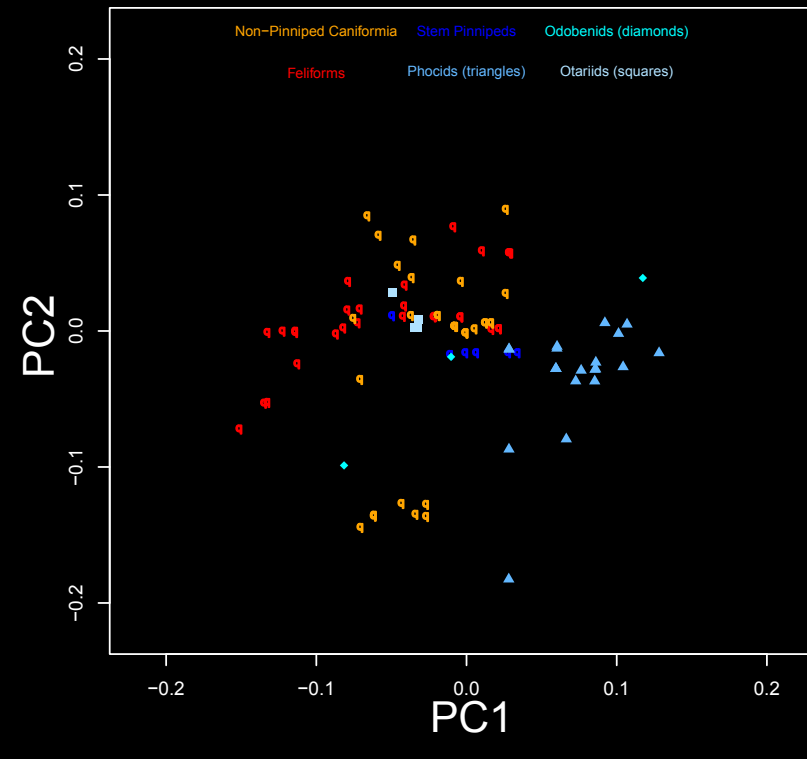

0Mya

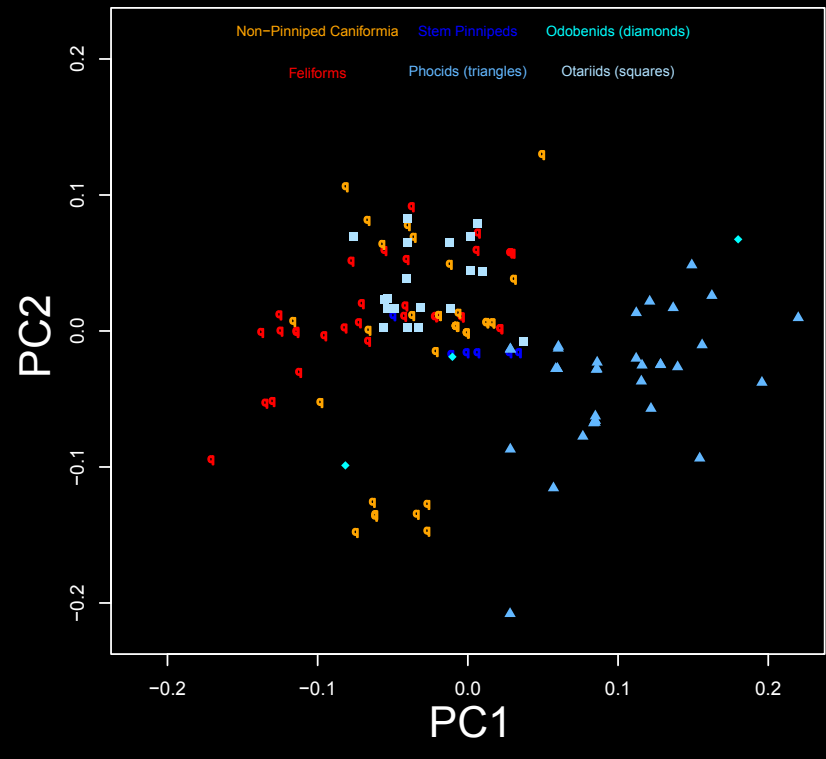

Supplement: Additional file 3: — IE_Evomorphospace_PC1PC2_otarioidea.pdf, figure, Evolutionary morphospace for PC1 vs PC2 for otarioidea. [file 12862_2015_285_MOESM3_ESM.pdf]

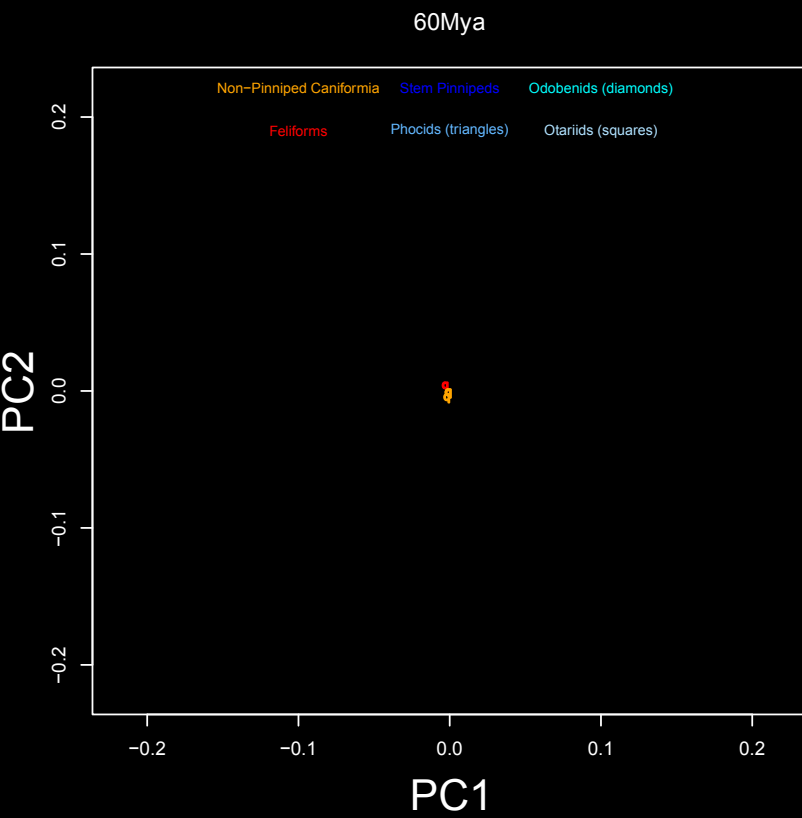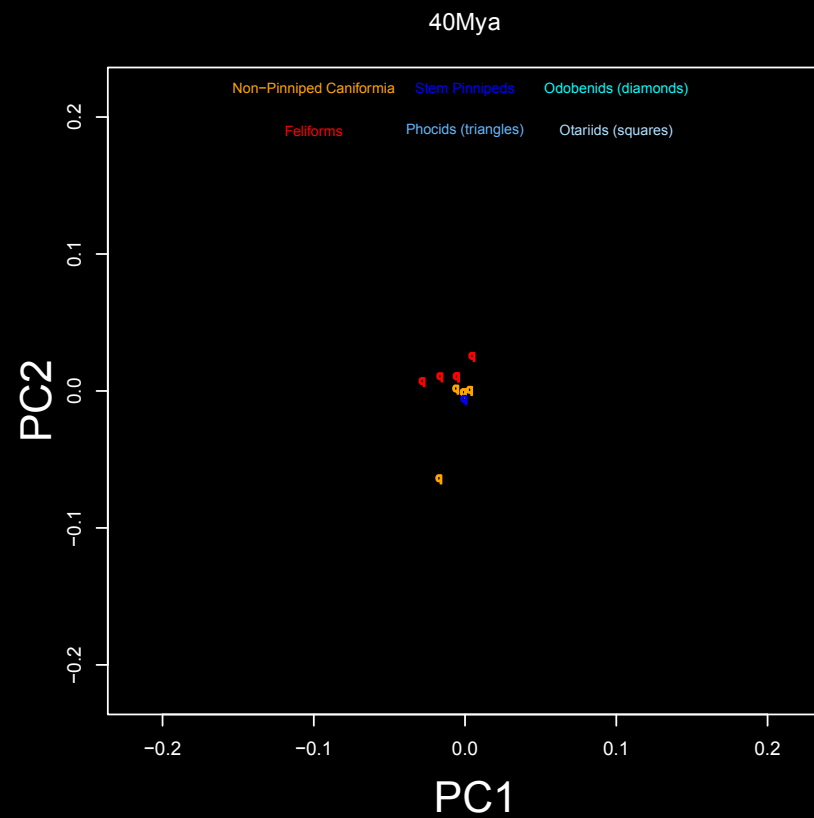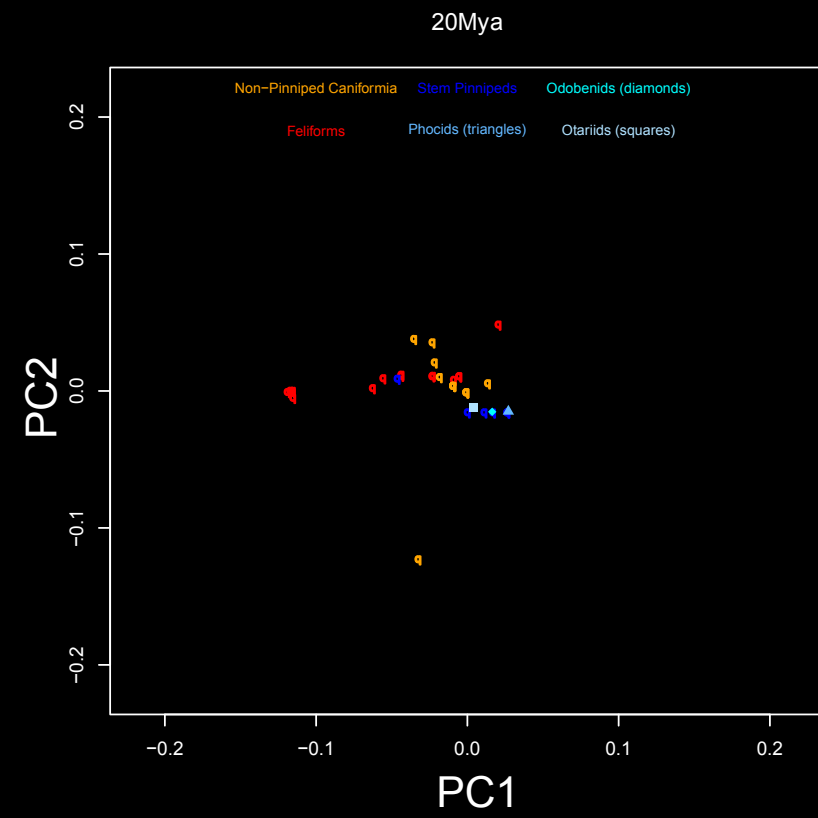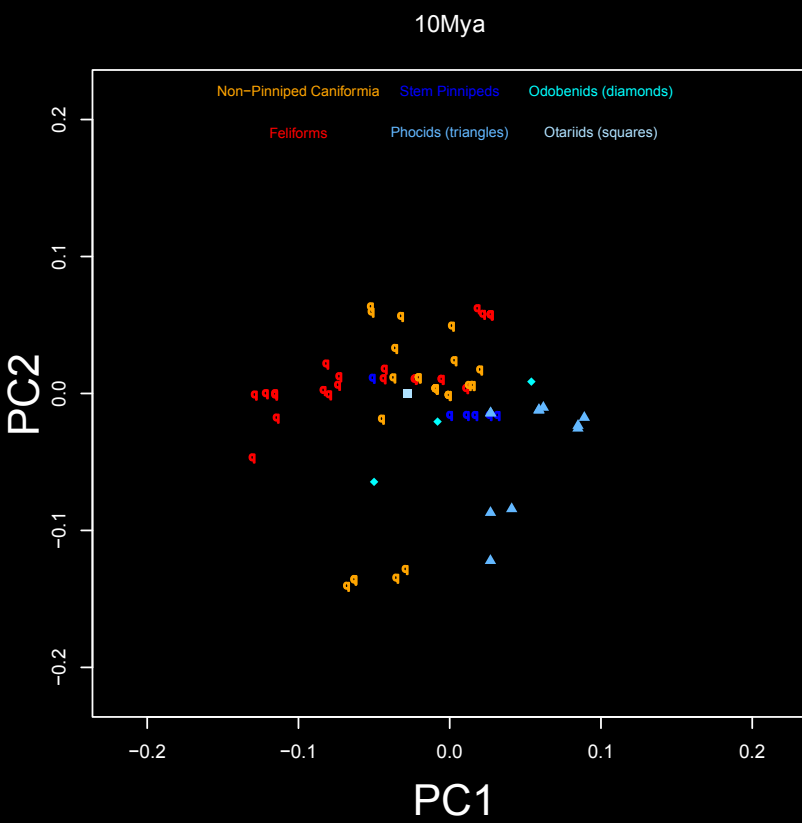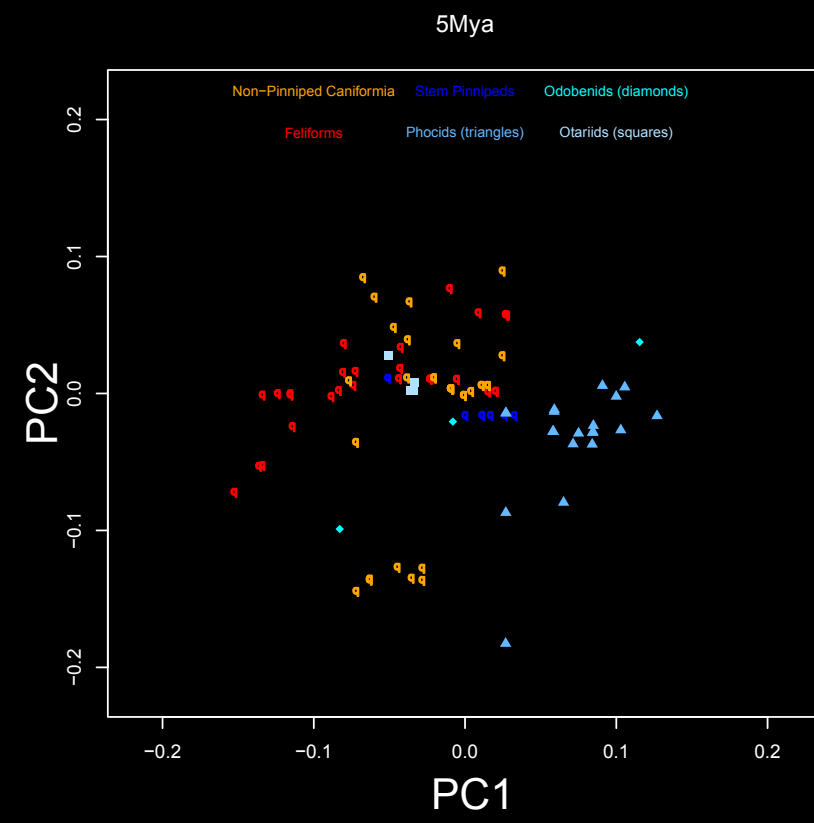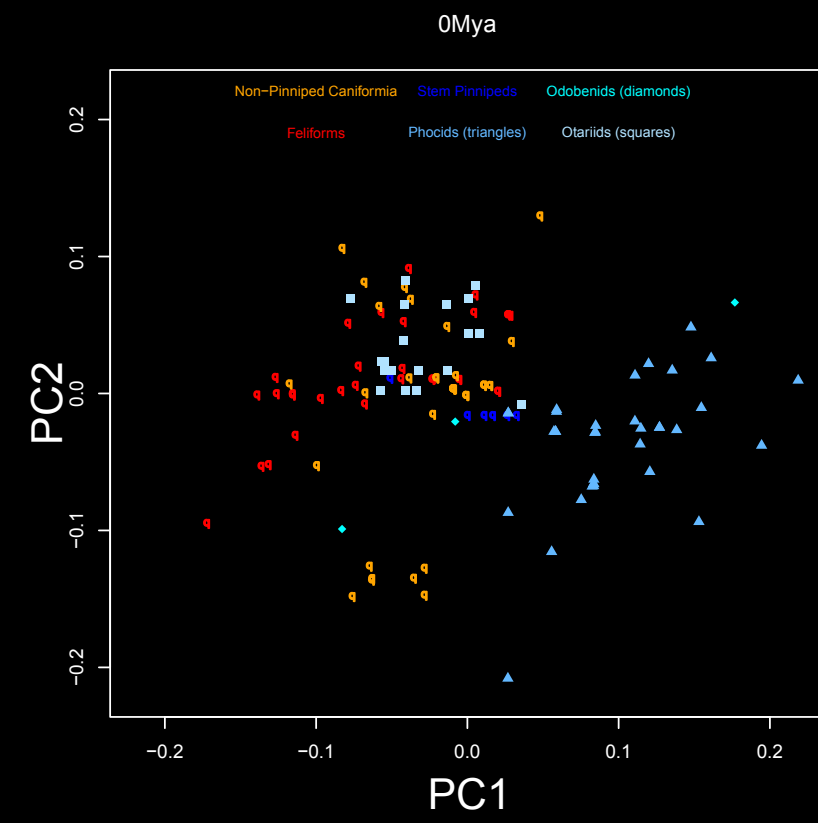

Supplement: Additional file 6: — IE_Evomorphospace_PC1PC2_phocomorpha.pdf, figure, Evolutionary morphospace for PC1 vs PC2 for phocomorpha. [file 12862_2015_285_MOESM6_ESM.pdf]

60Mya

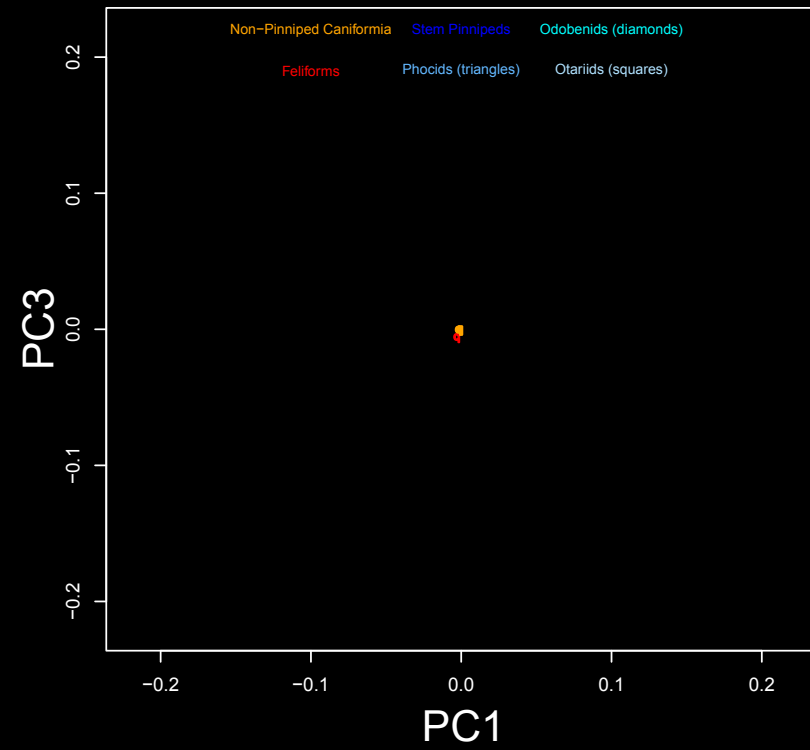

40Mya

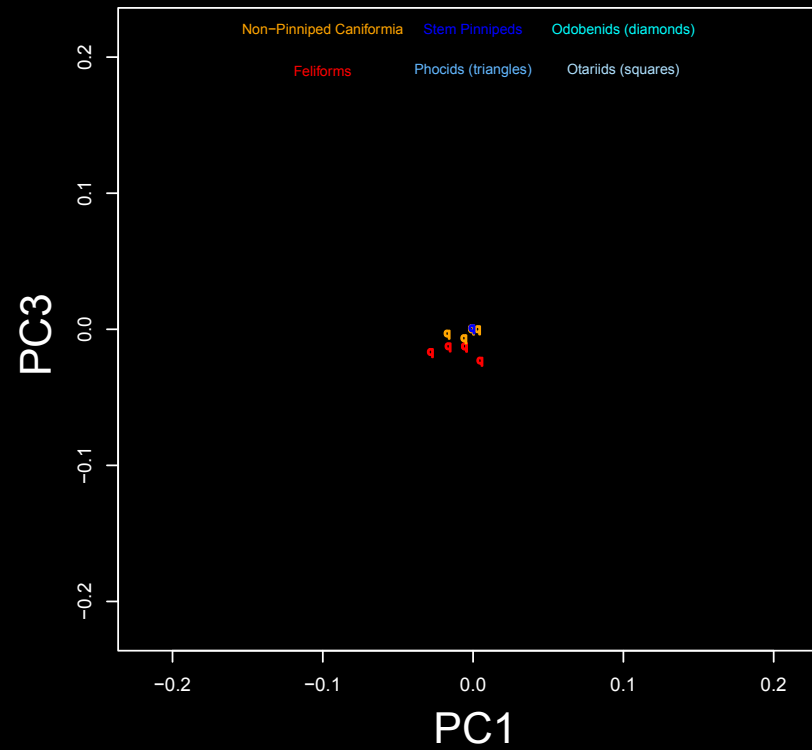

20Mya

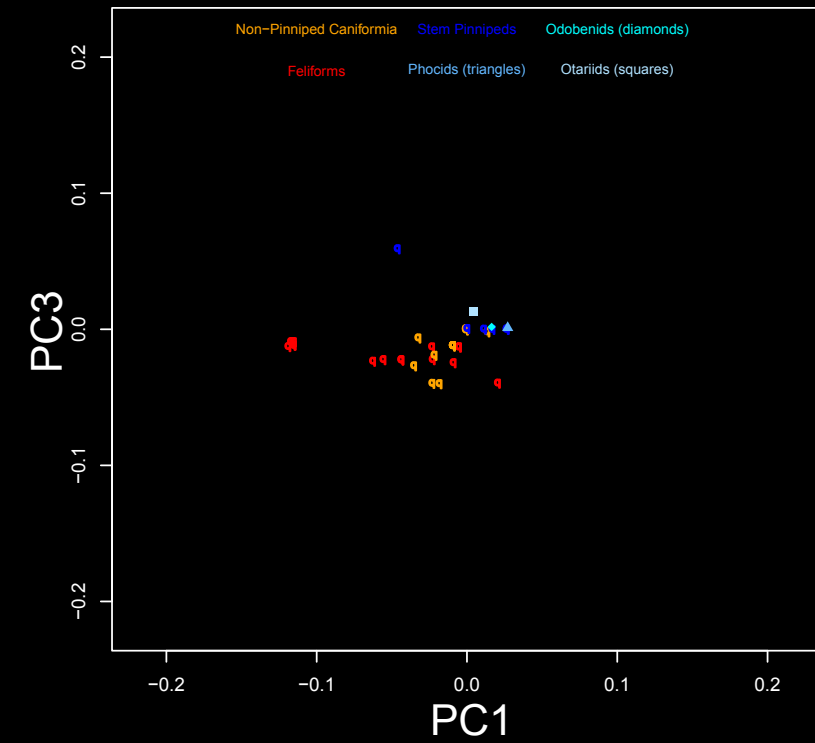

10Mya

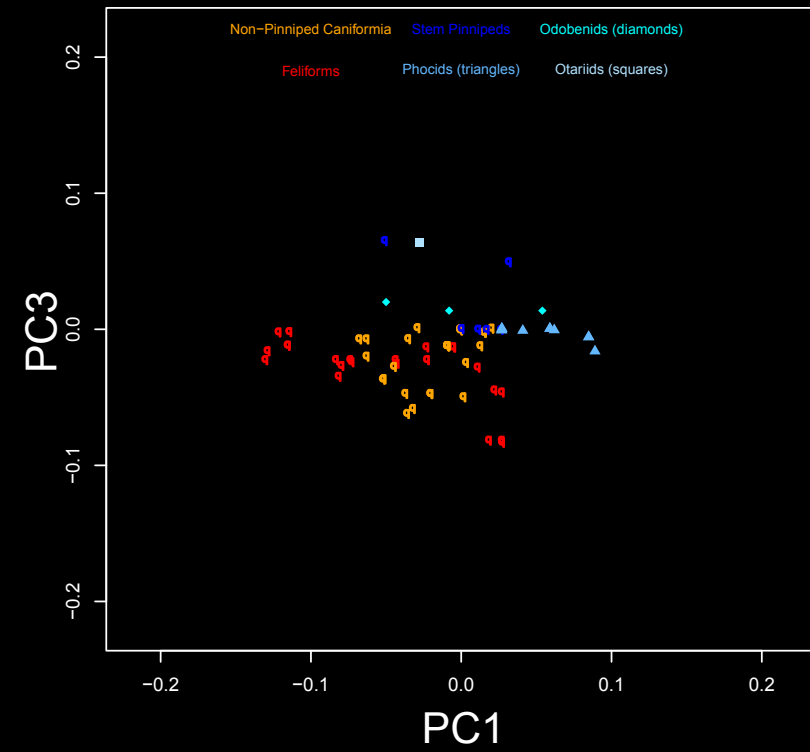

5Mya

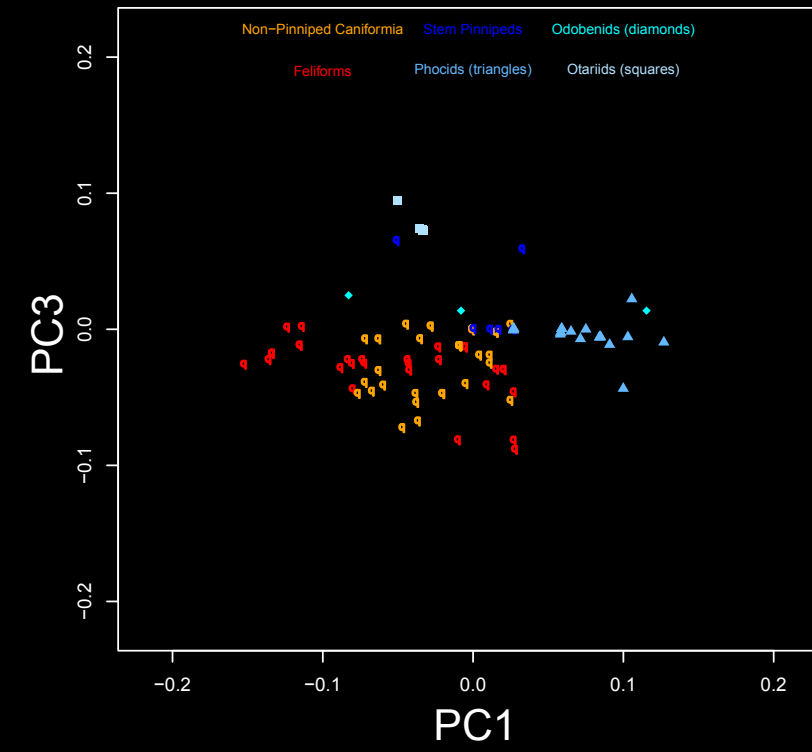

0Mya

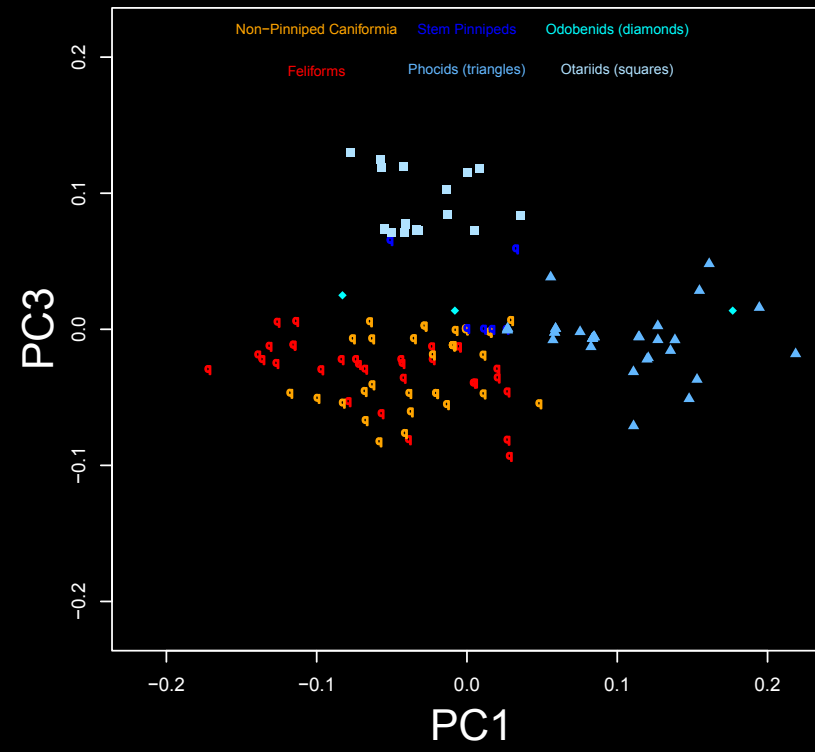

Supplement: Additional file 7: — IE_Evomorphospace_PC1PC3_phocomorpha.pdf, figure, Evolutionary morphospace for PC1 vs PC3 for phocomorpha. [file 12862_2015_285_MOESM7_ESM.pdf]

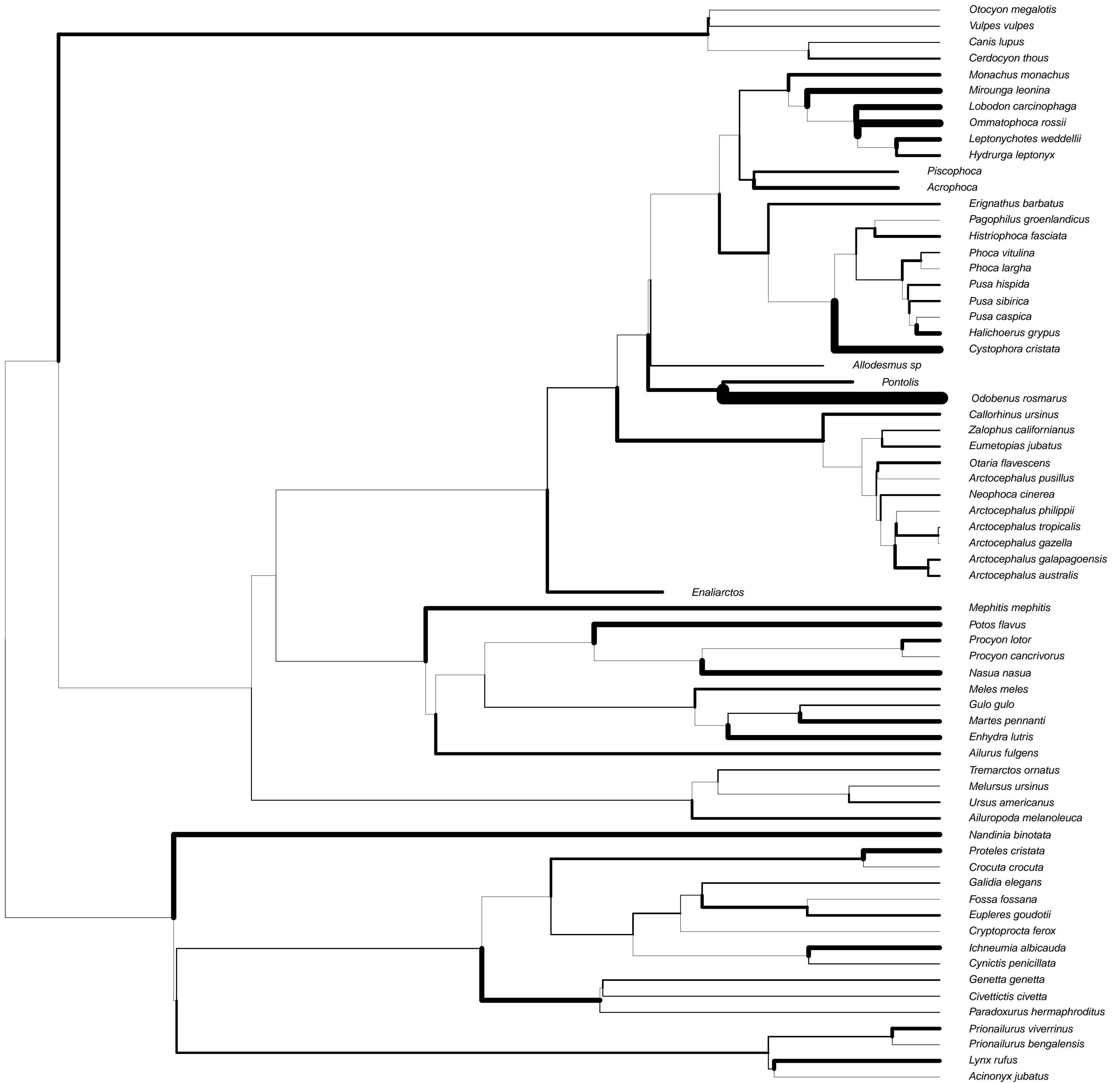

Supplement: Additional file 10: — MultivariateIE_phocomorpha.pdf, figure, Multivariate distances calculated from nodes from IE model. Thickness of branches represents multivariate distance between ancestor–descendant. [file 12862_2015_285_MOESM10_ESM.pdf]

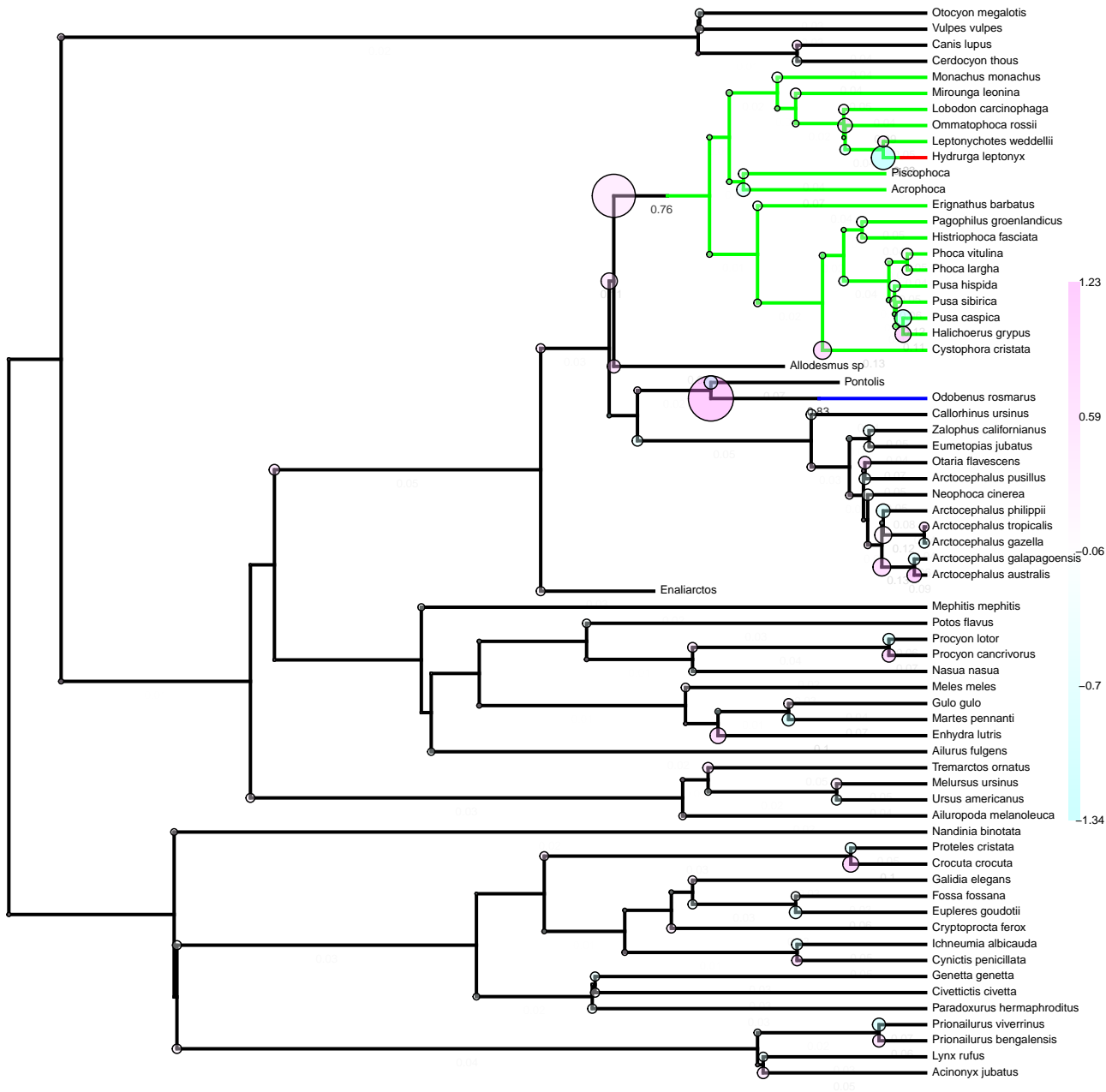

Supplement: Additional file 11: — Bayou_PC1_otarioidea.pdf, figure, Results of bayou analysis on PC1 for otarioidea. Circles at the node represent the likelihood value of a shift occurring at that node. Nodes with a likelihood value of 0.2 or greater were mapped with a regime shift, represented by a change in color, on the subsequent branches. [file 12862_2015_285_MOESM11_ESM.pdf]

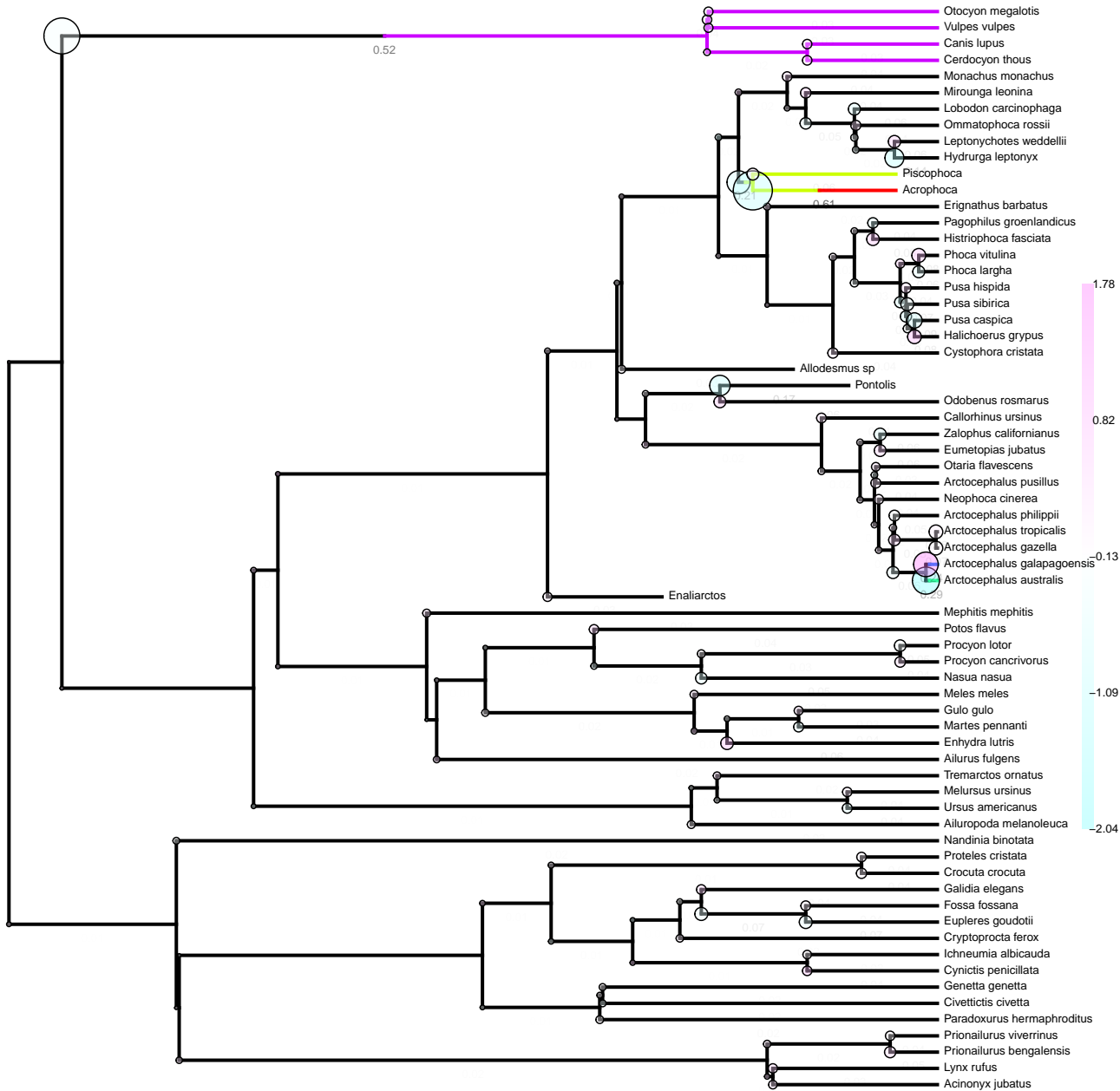

Supplement: Additional file 12: — Bayou_PC2_otarioidea.pdf, figure, Results of bayou analysis on PC2 for otarioidea. Circles at the node represent the likelihood value of a shift occurring at that node. Nodes with a likelihood value of 0.2 or greater were mapped with a regime shift, represented by a change in color, on the subsequent branches. [file 12862_2015_285_MOESM12_ESM.pdf]

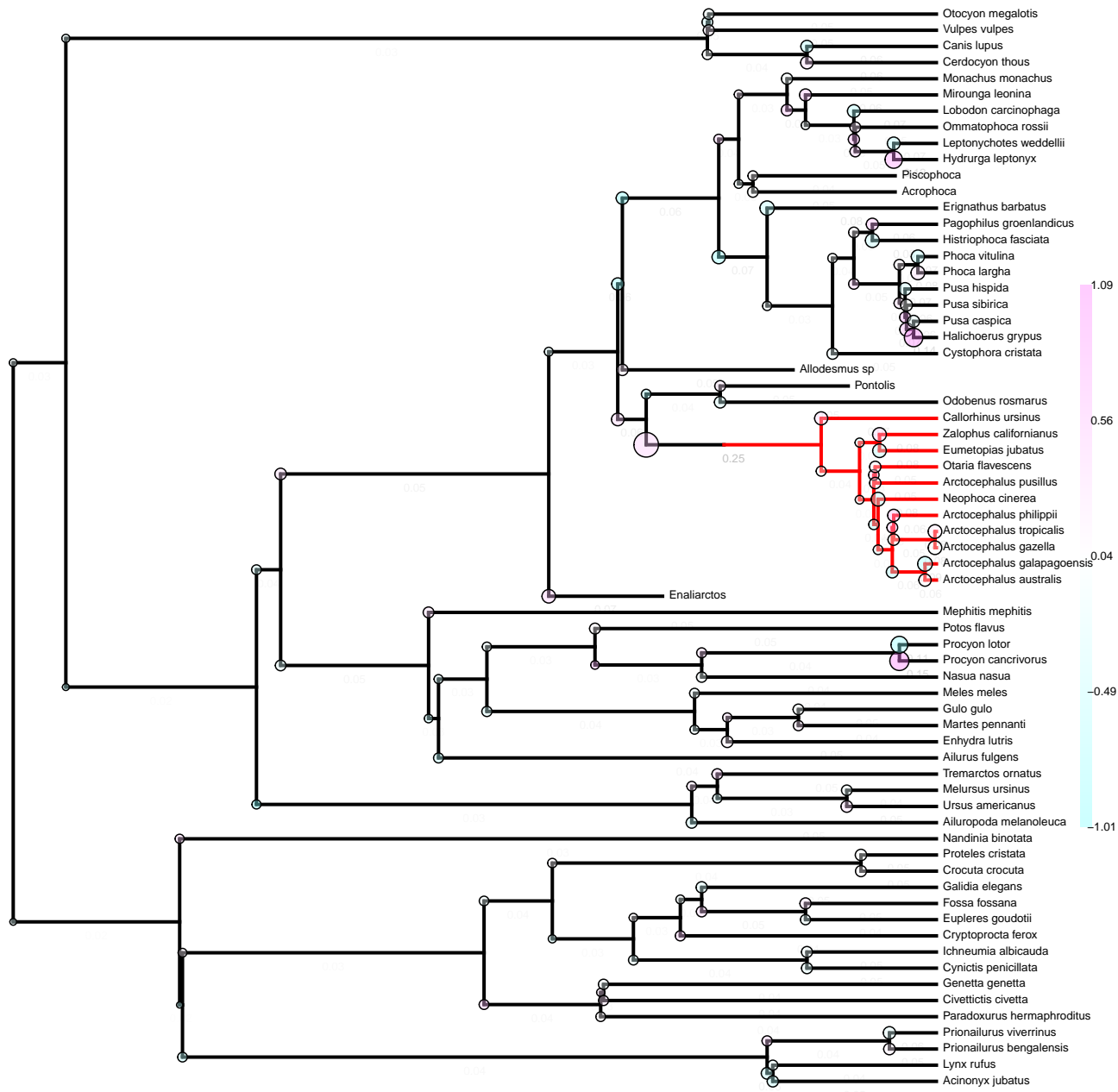

Supplement: Additional file 13: — Bayou_PC3_otarioidea.pdf, figure, Results of bayou analysis on PC3 for otarioidea. Circles at the node represent the likelihood value of a shift occurring at that node. Nodes with a likelihood value of 0.2 or greater were mapped with a regime shift, represented by a change in color, on the subsequent branches. [file 12862_2015_285_MOESM13_ESM.pdf]

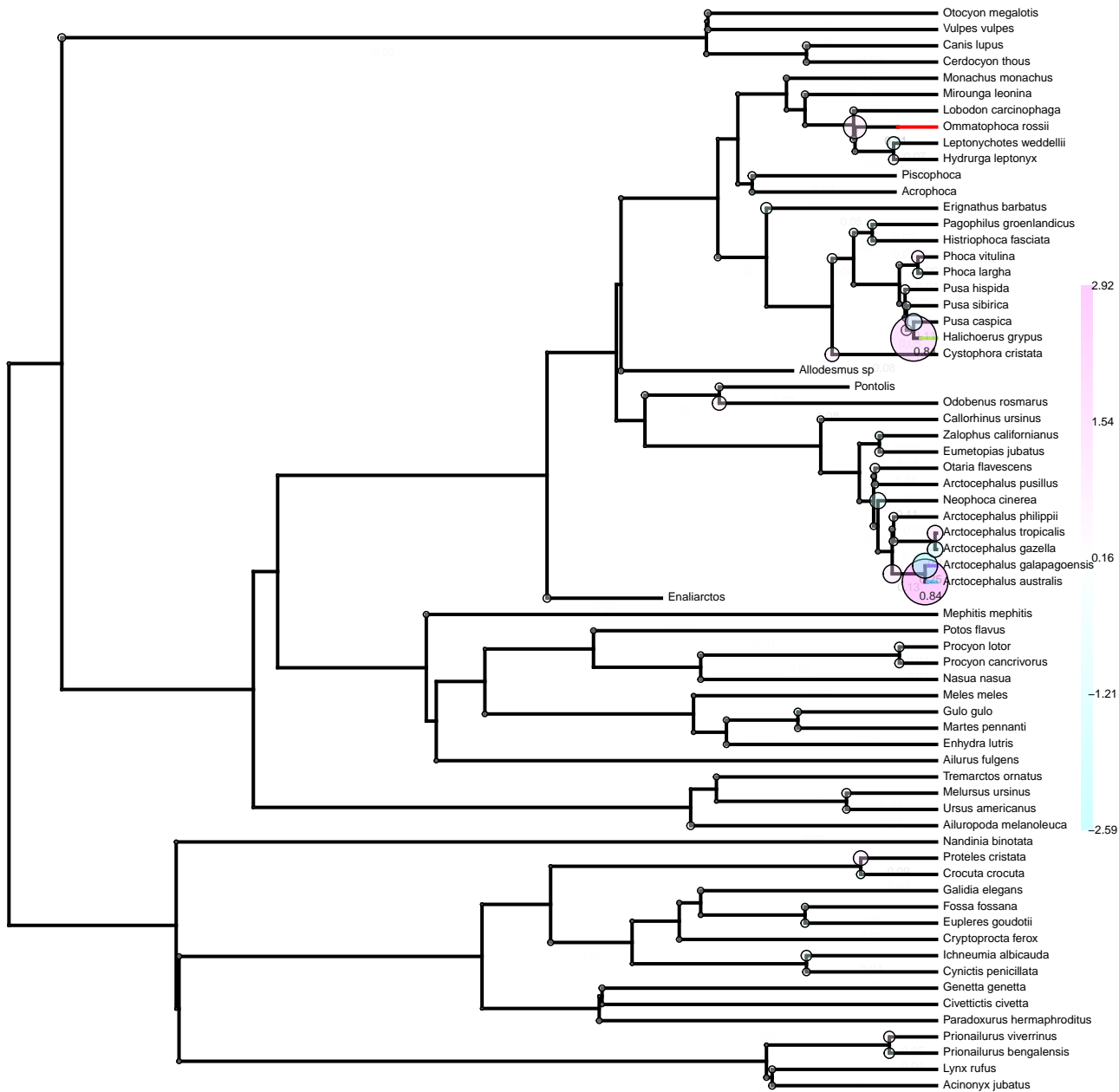

Supplement: Additional file 14: — Bayou_PC4_otarioidea.pdf, figure, Results of bayou analysis on PC4 for otarioidea. Circles at the node represent the likelihood value of a shift occurring at that node. Nodes with a likelihood value of 0.2 or greater were mapped with a regime shift, represented by a change in color, on the subsequent branches. [file 12862_2015_285_MOESM14_ESM.pdf]

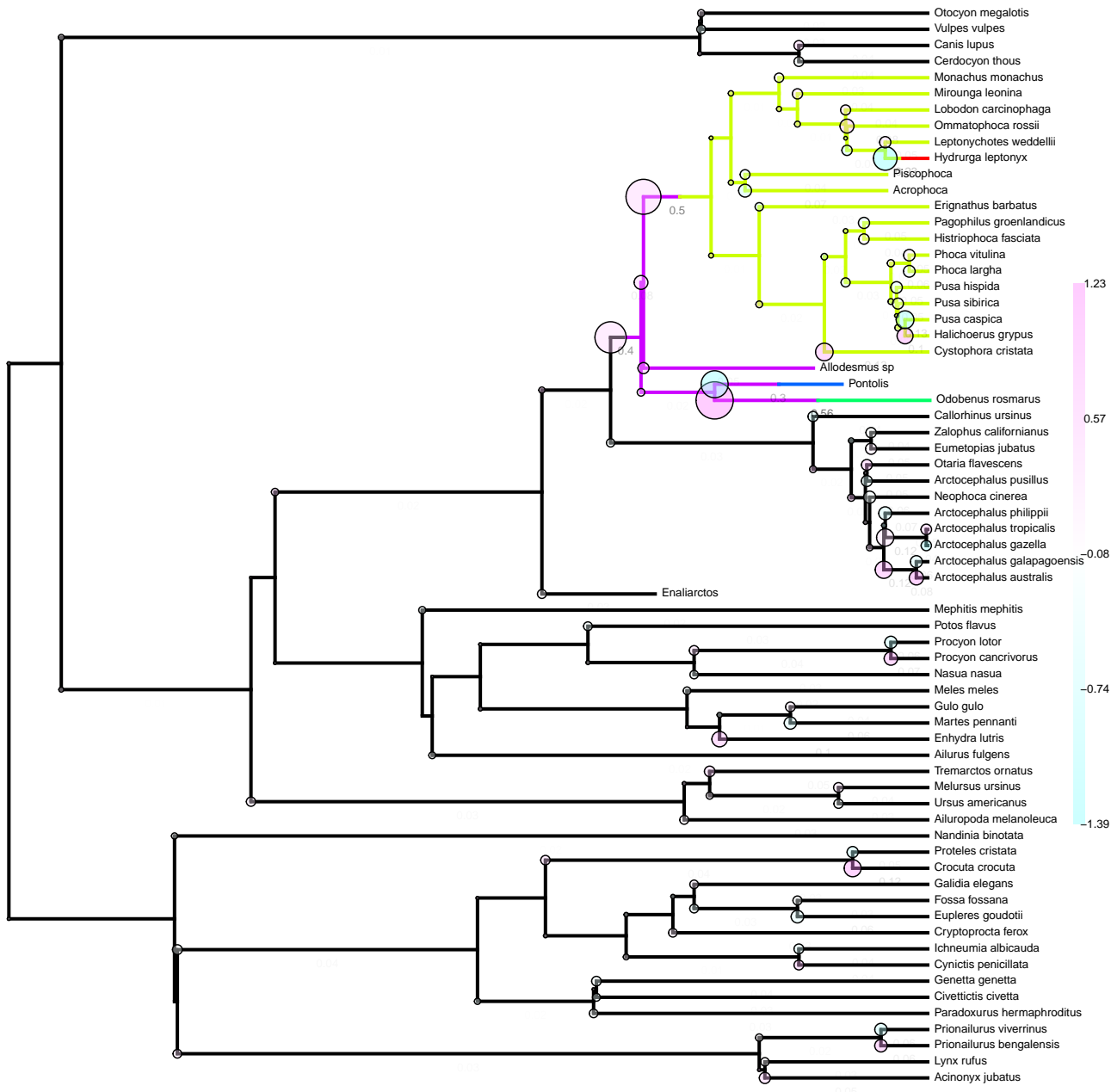

Supplement: Additional file 15: — Bayou_PC1_phocomorpha.pdf, figure, Results of bayou analysis on PC1 for phocomorpha. Circles at the node represent the likelihood value of a shift occurring at that node. Nodes with a likelihood value of 0.2 or greater were mapped with a regime shift, represented by a change in color, on the subsequent branches. [file 12862_2015_285_MOESM15_ESM.pdf]

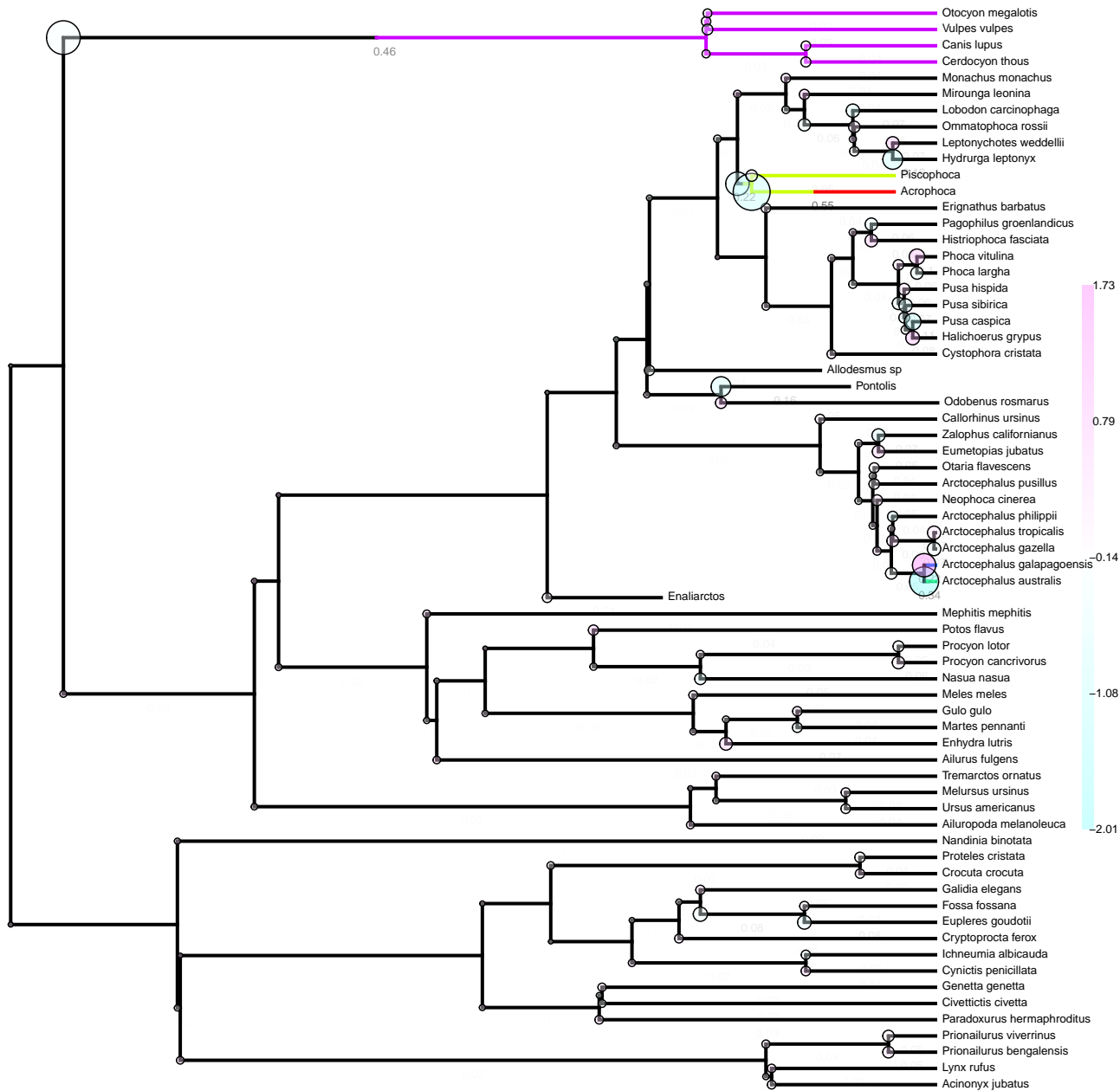

Supplement: Additional file 16: — Bayou_PC2_phocomorpha.pdf, figure, Results of bayou analysis on PC2 for phocomorpha. Circles at the node represent the likelihood value of a shift occurring at that node. Nodes with a likelihood value of 0.2 or greater were mapped with a regime shift, represented by a change in color, on the subsequent branches. [file 12862_2015_285_MOESM16_ESM.pdf]

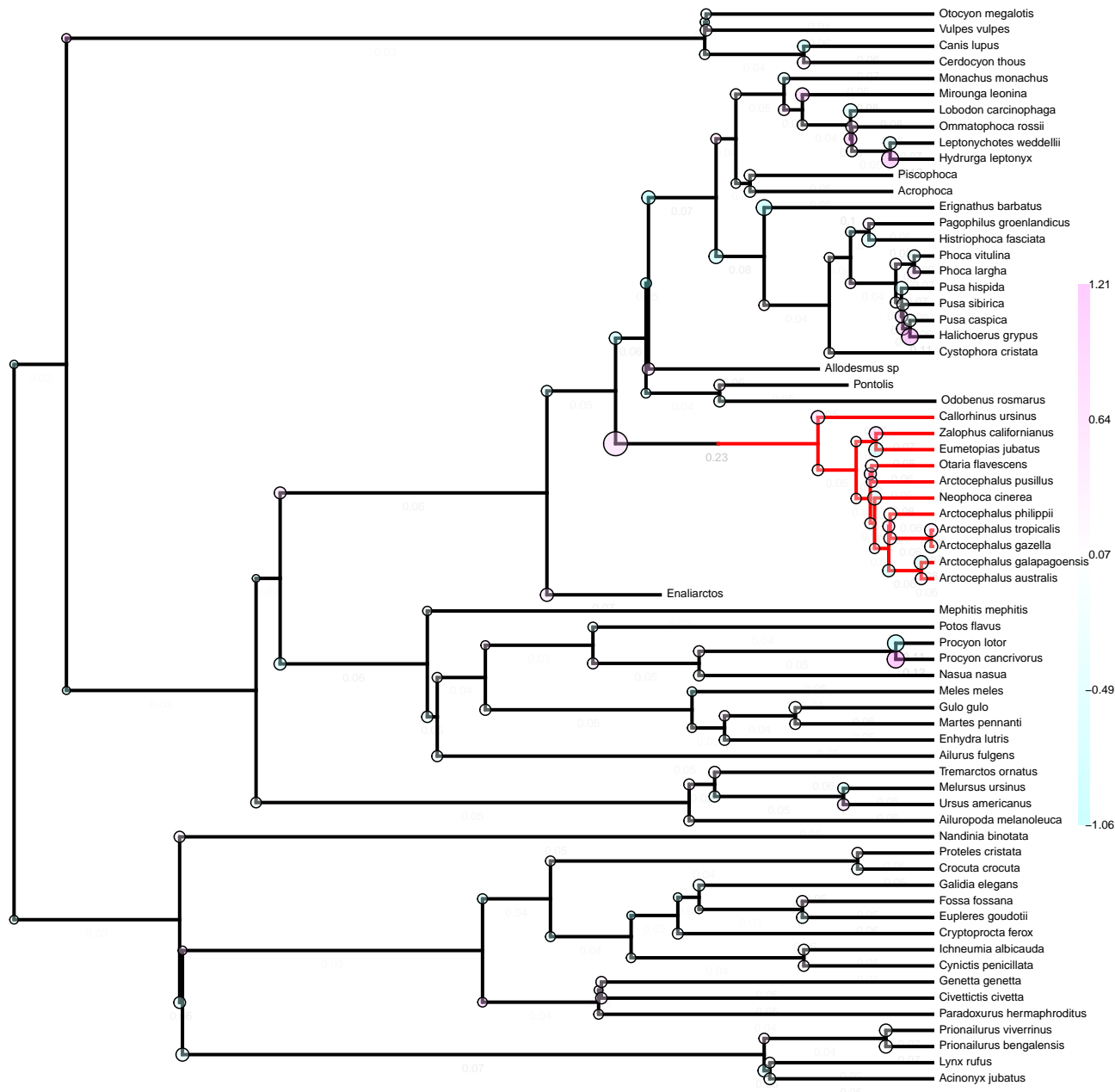

Supplement: Additional file 17: — Bayou_PC3_phocomorpha.pdf, figure, Results of bayou analysis on PC3 for phocomorpha. Circles at the node represent the likelihood value of a shift occurring at that node. Nodes with a likelihood value of 0.2 or greater were mapped with a regime shift, represented by a change in color, on the subsequent branches. [file 12862_2015_285_MOESM17_ESM.pdf]

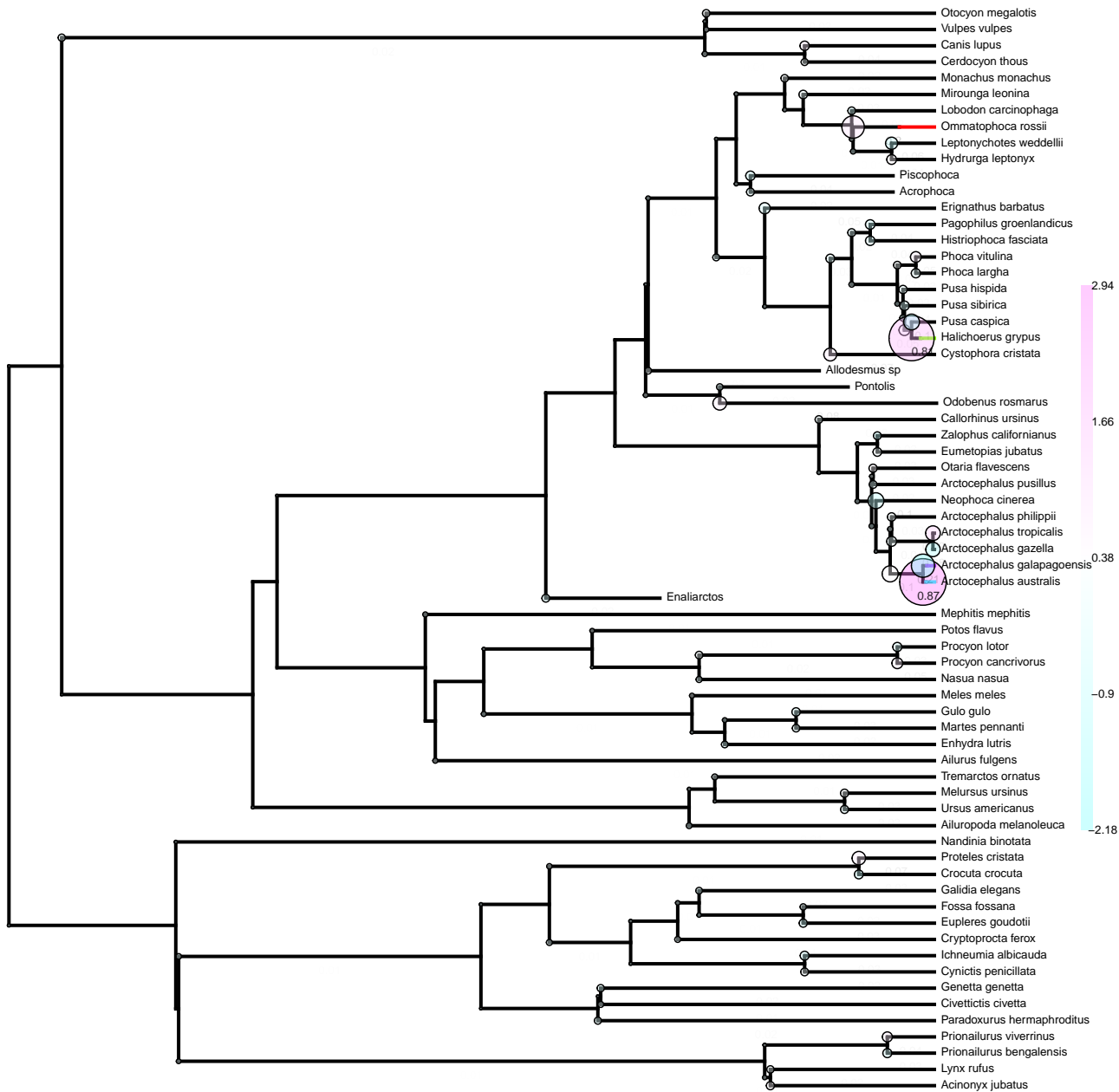

Supplement: Additional file 18: — Bayou_PC4_phocomorpha.pdf, figure, Results of bayou analysis on PC4 for phocomorpha. Circles at the node represent the likelihood value of a shift occurring at that node. Nodes with a likelihood value of 0.2 or greater were mapped with a regime shift, represented by a change in color, on the subsequent branches. [file 12862_2015_285_MOESM18_ESM.pdf]
